# Icaritin plus TACE improves survival in advanced HCC with macrovascular invasion: a multicenter cohort study

**DOI:** 10.3389/fimmu.2026.1684486

**Published:** 2026-05-29

**Authors:** Huilin Lu, Tao Xu, Jing Li, Bufu Tang, Yulan Zeng, Xinghai Li, Jiangping Cun, Xiangwen Xia, Jihong Hu, Xuancheng Xie, Hongjie Fan

**Affiliations:** 1 Department of Interventional Therapy, Xinxiang Central Hospital / the Fourth Clinical College of Xinxiang Medical University, Xinxiang, Henan, China; 2 Department of Radiology, Union Hospital, Tongji Medical College, Huazhong University of Science and Technology, Wuhan, China; 3 Hubei Provincial Clinical Research Center for Precision Radiology & Interventional Medicine, Wuhan, China; 4 Hubei Provincial Key Laboratory of Molecular Imaging, Wuhan, China; 5 Department of Radiation Oncology, Zhongshan Hospital, Fudan University, Shanghai, China; 6 Cancer Center, Union Hospital, Tongji Medical College, Huazhong University of Science and Technology, Wuhan, China; 7 Department of Minimally Invasive Intervention, Ganzhou People's Hospital, Ganzhou, China; 8 Department of Radiology, the Second Affiliated Hospital of Kunming Medical University, Kunming, China; 9 Department of Interventional Radiology, The First Affiliated Hospital of Kunming Medical University, Kunming, China; 10 Department of Radiology, The First People's Hospital of Yunnan Province, Kunming University of Science and Technology Affiliated Hospital, Kunming, China

**Keywords:** hepatocellular carcinoma, macrovascular invasion, survival outcomes, transarterial chemoembolization, tumor response

## Abstract

**Background:**

Icaritin, a prenylated flavonoid extracted from *Epimedium*, induces apoptosis and modulates immunity with proven antitumor activity in hepatocellular carcinoma (HCC). This study aimed to compare survival outcomes of transarterial chemoembolization (TACE) plus Icaritin versus TACE alone in HCC patients with macrovascular invasion (MVI).

**Methods:**

This multicenter retrospective cohort study, conducted from October 2022 to June 2025, included 288 patients with HCC and MVI from five tertiary hospitals. Patients were assigned to either the TACE plus Icaritin group (n = 144) or the TACE monotherapy group (n = 144). Tumor response was evaluated by mRECIST on contrast-enhanced CT/MRI. Overall survival and progression-free survival were estimated using Kaplan Meier curves and compared by log-rank test; prognostic factors were identified via Cox regression.

**Results:**

The Icaritin–TACE group showed significantly better outcomes, with longer median OS (16.3 vs. 13.3 months; P = 0.020) and PFS (8.5 vs. 7.6 months; P = 0.006) than the TACE monotherapy group. The disease control rate (DCR) was also higher in the Icaritin–TACE group (84.0% vs. 72.2%; P = 0.015). Multivariate Cox regression analysis identified ECOG performance status, alpha-fetoprotein (AFP) levels, number of lesions, and maximum lesion diameter as independent predictors of OS, while lesion count was independently associated with PFS. The overall incidence of any grade adverse events was similar between groups.

**Conclusion:**

Icaritin combined with TACE improves OS, PFS, and DCR compared to TACE alone in patients with MVI-associated HCC.

## Introduction

Hepatocellular carcinoma (HCC) remains among the most prevalent cancers worldwide and is the third leading cause of cancer-related death (1). Although surgical resection offers curative potential—particularly in patients classified as BCLC 0 or A—the prognosis worsens considerably once macroscopic vascular invasion (MVI) occurs (2, 3). These patients typically fall into BCLC stage C, yet those with preserved hepatic function and patent portal veins may still benefit from transarterial chemoembolization (TACE) combined with systemic therapies, as supported by multiple studies (4–7). Notably, MVI has emerged as an independent predictor of poor outcome after TACE (HR = 1.6) (7), and adding sorafenib has significantly extended time to progression (P = 0.011) (4). Moreover, recent trials indicate that combining TACE with immune checkpoint inhibitors (ICIs) and lenvatinib can meaningfully improve overall survival (OS) (8), findings further validated by meta-analyses (9). Although treatment advances such as TACE combined with targeted agents and immunotherapies have demonstrated improved survival, optimizing therapeutic regimens for advanced HCC with MVI continues to be a critical priority tailored to patient liver function and tumor burden.

Icaritin, a new small-molecule flavonoid immunomodulator derived from the traditional Chinese herb *Epimedii Folium*, has shown promising results in advanced HCC (10–12). Preclinical work reveals that it induces immunogenic cell death (ICD) through mitochondrial autophagy and apoptosis *in vitro* and *in vivo (*10, 12). It also appears to enhance antitumor immunity by inhibiting IL-6/JAK/STAT3 signaling, reducing cytokines and immune checkpoint expressions, and suppressing myeloid-derived suppressor cells (11). A multicenter, single-arm, open-label phase II study revealed that in patients with advanced HCC treated with Icaritin, elevated serum alpha-fetoprotein (AFP) levels and a higher composite Th1/Th2 cytokine score were significantly associated with improved survival outcomes, suggesting Icaritin as a promising alternative immunotherapeutic agent (13). Subsequently, a randomized, double-blind phase III trial showed that Icaritin significantly prolonged overall survival (median OS 13.54 months) in a prospectively enriched subgroup of HBV-related advanced HCC patients with poor performance status who were positive for a composite biomarker score (CBS)—defined by meeting at least two of the following criteria: serum AFP ≥400 ng/mL, TNF-α <2.5 pg/mL, and IFN-γ ≥7.0 pg/mL—while maintaining a favorable safety profile (14). This favorable data prompted approval by the China National Medical Products Administration in 2022 (15). That same year, leading hepatology and oncology experts in China included Icaritin in the revised 2022 edition of the *Guidelines for the Diagnosis and Treatment of Primary Liver Cancer*, recommending its use in the treatment of advanced HCC (Evidence Level 2, Recommendation Grade B) (16).

On the strength of this evidence, we conducted a multicenter retrospective study to assess whether adding Icaritin to TACE confers additional survival benefits in high-risk HCC patients with MVI, providing real-world support for integrating traditional immunomodulatory therapies into modern oncological care strategies.

## Material and methods

### Study design and participants

This multicenter, retrospective cohort study was conducted across five tertiary medical centers in China between October 2022 and June 2025 to assess the therapeutic efficacy of combining Icaritin with TACE versus TACE alone in patients diagnosed with HCC complicated by MVI. The study protocol received institutional ethics approval (IRB No. IEC-2026-0472), and all clinical procedures adhered to the ethical standards set forth in the Declaration of Helsinki. The need for informed consent was waived by the committee as only de-identified retrospective data were analyzed. This cohort study has been reported in line with the STROCSS guidelines (17).

Eligible participants were aged 18 to 80 years and had either histologically or imaging-confirmed MVI, with limited portal vein tumor thrombus (PVTT). Inclusion criteria further required an Eastern Cooperative Oncology Group (ECOG) performance score of 0–1, Child–Pugh class A or B hepatic function, and a life expectancy exceeding three months.

**Figure 1:**
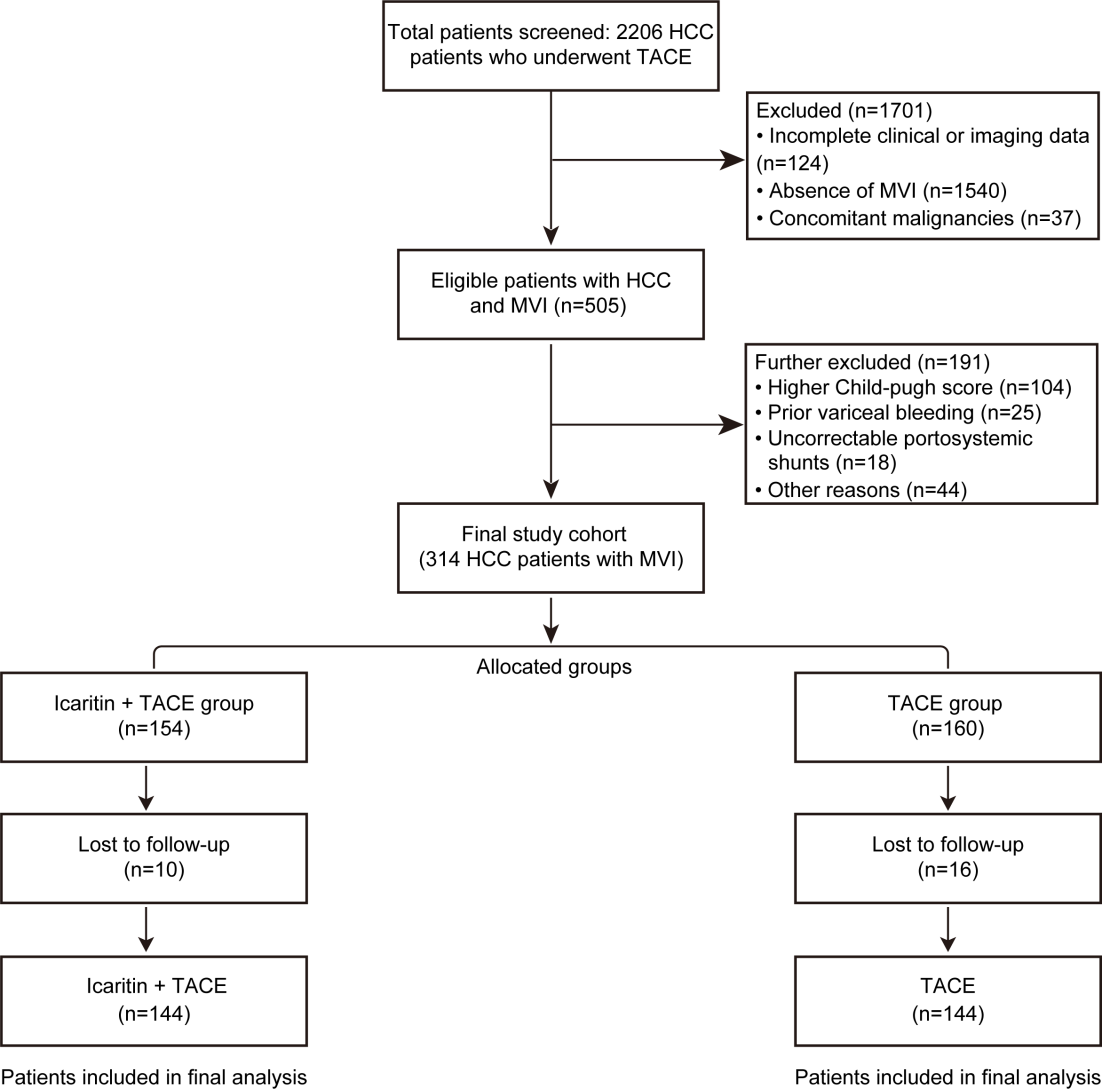
Patient screening, eligibility, and analysis flow diagram. HCC, hepatocellular carcinoma; MVI, macrovascular invasion; TACE, transarterial chemoembolization.

Patients were excluded if they had other concurrent malignancies, extensive PVTT or compromised portal flow not suitable for embolization, coagulation dysfunction with a prothrombin time exceeding 6 seconds above the normal range, hepatic encephalopathy, refractory ascites, prior variceal bleeding, uncorrectable portosystemic shunts, active infections, or incomplete clinical/imaging records. Treatment allocation was not randomized. Icaritin was prescribed according to local institutional practice and physician assessment after evaluating liver function, performance status, and overall clinical suitability. Patients in the comparator group received TACE alone because Icaritin was not initiated in their treatment course for clinical, patient-related, or access-related reasons. The patient selection process is illustrated in **Figure 1**. In total, 288 patients were included in the final analysis, with 144 patients in the TACE-alone group and 144 patients in the TACE plus Icaritin group.

### TACE procedure

Before treatment, patients underwent full laboratory evaluations—including blood counts, liver and renal function tests, and coagulation profiles—along with contrast-enhanced CT or MRI to assess tumor burden, vascular anatomy, and portal vein patency. Treatment eligibility was reconfirmed based on ECOG performance status and Child–Pugh classification. Written informed consent was obtained after patients were thoroughly counseled on risks, benefits, and treatment alternatives.

TACE procedures were performed by interventional radiologists with over eight years of experience. Under sterile conditions and local anesthesia (1% lidocaine), femoral artery access was obtained using the Seldinger technique. A 5 Fr vascular sheath and Yashiro catheter were used to map the celiac trunk and superior mesenteric artery. A 2.7 Fr Progreat^®^ microcatheter was then superselectively advanced into tumor-feeding subsegmental arteries. A chemotherapeutic emulsion—doxorubicin or cisplatin mixed with iodized oil, or drug-eluting beads—was infused until near-stasis, followed by gelatin sponge particles to ensure complete embolization. All devices were removed post-procedure, and hemostasis was achieved by compressing the femoral site for 6–8 hours. Supportive care included IV fluids and symptomatic management with analgesics and antiemetics as needed.

### Systemic therapy protocol

Oral Icaritin (600 mg, twice daily post-meal) was initiated within 3–5 days after the first TACE procedure, and this timing, dosage, and administration schedule were consistent across all participating centers. Dose adjustments, temporary suspension, or discontinuation were made if liver function abnormalities occurred based on clinical assessment. Patients receiving or requiring systemic targeted therapies—such as lenvatinib, donafenib, regorafenib, sorafenib, or apatinib—could continue or initiate such treatments according to individual clinical indications.

### Follow-up protocol

Patients underwent contrast-enhanced CT or MRI 4 to 6 weeks after the initial procedure to assess treatment response, following the modified Response Evaluation Criteria in Solid Tumors (mRECIST). Each evaluation was independently conducted by two board-certified radiologists with more than 10 years of experience in hepatic imaging. Both readers were blinded to treatment allocation and clinical outcomes. In cases of discrepant assessments, consensus was reached through joint review. If no contraindications were present, additional TACE sessions were administered in cases of persistent viable tumor tissue. Serum AFP levels were monitored every 1 to 2 months, while surveillance imaging—including ultrasound or contrast-enhanced CT/MRI—was performed at intervals of no more than 3 months to detect recurrence. OS was defined as the duration from the date of the first TACE (with or without Icaritin) until death from any cause or the date of the last follow-up. PFS was measured from the initiation of treatment to the first radiologic evidence of disease progression or death. The objective response rate (ORR) was calculated as the proportion of patients achieving either complete or partial tumor regression, while the disease control rate (DCR) included complete response, partial response, and stable disease. The primary endpoint was PFS, and the secondary endpoints included OS, ORR, and DCR.

### Statistical analysis

Data analysis was performed using Stata version 17.0 (StataCorp, College Station, TX, USA), SPSS version 27.0 (IBM Corp., Armonk, NY, USA) and R version 4.5.1. Missing data were rare (all variables <5% missing). Primary analyses used complete-case analysis; subjects with missing values on covariates required for a given analysis were excluded from that analysis. Continuous variables, such as patient age and tumor dimensions, were presented as mean ± standard deviation or median with interquartile range and compared using either unpaired two-sample t-tests or Mann–Whitney U tests, depending on data distribution. Categorical variables were expressed as frequencies and percentages, with between-group comparisons conducted using Pearson’s chi-square test or Fisher’s exact test where appropriate. Inter-reader reliability for mRECIST classifications was quantified using Cohen’s κ coefficient. For time-to-event analyses, proportional hazards assumptions were formally tested using Schoenfeld residuals for each Cox proportional hazards model. No significant violations of the PH assumption were detected (global test P > 0.10). Propensity scores matching were estimated with a logistic regression model including all prespecified baseline covariates (listed in **Table 1**). We conducted 1:1 nearest-neighbor matching without replacement using a caliper of 0.2 × SD (logit [PS]). Covariate balance before and after matching was assessed by standardized mean differences (SMD), with SMD < 0.1 indicating acceptable balance. Survival outcomes (OS and PFS) were estimated using the Kaplan–Meier method and differences between groups were assessed via the log-rank test. Candidate predictors were first screened using the Boruta algorithm. Variables classified as Unimportant were excluded from the primary multivariable Cox regression to reduce model complexity and risk of overfitting. As a sensitivity analysis, we also fitted a full multivariable model including all covariates identified in univariable analyses and all were included in the multivariate analysis. Multicollinearity among candidate covariates was assessed using variance inflation factors (VIF). Subgroup analyses for OS and PFS were performed by fitting multivariable Cox proportional hazards models that included the treatment indicator, the subgroup variable and a treatment-by-subgroup interaction term. Interaction p-values were obtained using the Wald test for the interaction coefficient. All statistical tests were two-sided, and a threshold of P < 0.05 was used to define statistical significance.

**Table 1:** Comparison of baseline characteristics between patients treated with icaritin-TACE vs TACE alone.

	Icaritin-TACE group (n=144)	TACE alone group (n=144)	Total (n=288)	*P*
Gender, n (%)				0.195
Male	124 (86.1)	131 (91.0)	255 (88.5)	
Female	20 (13.9)	13(9.0)	33 (11.5)	
Age, median (IQR), yrs	55 (17)	56 (17)	56 (17)	0.807
ECOG score, n (%)^a^				0.704
0	100 (69.4)	97 (67.4)	197 (68.4)	
1	44 (30.6)	47 (32.6)	91 (31.6)	
Child-Pugh grade, n (%)				0.866
Grade A	123 (85.4)	122 (84.7)	245 (85.1)	
Grade B	21 (14.6)	22 (15.3)	43 (14.9)	
Targeted therapy, n (%)				0.719
None	37 (25.7)	42 (29.2)	79 (27.4)	
Lenvatinib	63 (43.8)	56 (38.9)	119 (41.3)	
Donafenib	38 (26.4)	37 (25.7)	75 (26.0)	
Regorafenib	6 (4.2)	9 (6.3)	15 (5.2)	
Sessions of TACE, n (%)				0.619
1	29 (20.2)	36 (25.0)	65 (22.6)	
2	43 (29.9)	44 (30.6)	87 (30.2)	
≥3	72 (50.0)	64 (44.4)	136 (47.2)	
Viral infection, n (%)				0.281
Hepatitis B	138 (95.8)	134 (93.1)	272 (94.4)	
Hepatitis C	5 (3.5)	7 (4.9)	12 (4.2)	
Other	1 (0.7)	3 (2.1)	4 (1.4)	
Portal vein tumor thrombus, n (%)				0.673
None	87 (60.4)	70 (48.6)	157 (54.5)	
Type I	21 (14.6)	21 (14.6)	42 (14.6)	
Type II	29 (20.1)	43 (29.9)	72 (25.0)	
Type III	6 (4.2)	9 (6.3)	15 (5.2)	
Type IV	1 (0.7)	1 (0.7)	2 (0.7)	
Ascites, n (%)^b^				0.086
None	138 (95.8)	132 (91.7)	270 (93.8)	
Grade 1	6 (4.2)	9 (6.3)	15 (5.2)	
Grade 2	0 (0.0)	3 (2.1)	3 (1.0)	
Albumin, mean (SD), g/L	36.2 (±4.8)	35.3 (±4.6)	35.8 (±4.7)	0.105
Functional parameters, median (IQR)				
ALT, U/L	54.0 (72.8)	59.0 (65.8)	55.5 (74.0)	0.676
AST, U/L	74.5 (95.0)	60.5 (82.8)	68.5 (85.3)	0.417
ALP, U/L	144.5 (129.3)	167.0 (120.5)	153.5 (124.3)	0.228
GGT, U/L	138.5 (165.0)	131.0 (156.3)	133.0 (160.0)	0.801
Platelets,×10⁹/L	127.0 (87.5)	130.0 (105.3)	128.5 (93.8)	0.362
PT, s	12.9 (1.9)	13.1 (2.3)	13.0 (2.1)	0.412
Total bilirubin, mg/dL	21.100 (13.7)	20.200 (16.6)	20.900 (15.9)	0.742
AFP, median (IQR), ng/mL	163.625 (990.7)	58.915 (903.9)	133.285 (992.8)	0.130
Extrahepatic metastases, n (%)^c^				0.235
No	137 (95.1)	132 (91.7)	269 (93.4)	
Yes	7 (4.9)	12 (8.3)	19 (6.6)	
Number of lesions, n (%)				0.238
≤3	45 (31.250)	36 (25.0)	81 (28.1)	
>3	99 (68.8)	108 (75.0)	207 (71.9)	
Maximum diameter of lesion, median (IQR), cm	6.200 (6.7)	6.400 (6.5)	6.250 (6.6)	0.960

Icaritin -TACE, transarterial chemoembolization plus Icaritin; TACE, transarterial chemoembolization; ECOG, Eastern Cooperative Oncology Group; ALT, alanine aminotransferase; AST, aspartate aminotransferase; ALP, Alkaline Phosphatase; GGT, gamma-glutamyl transferase; PT, prothrombin time (international ratio); AFP, alpha-Fetoprotein. ^a^ ECOG score of 0 indicates that patient is fully active and able to carry on all pre-disease activities without restriction, and 1 indicates that patient is restricted in physically strenuous activity but is ambulatory and able to carry out work of a light nature, including self-care. ^b^ Grade 1 indicates patients with mild ascites; Grade 2 indicates patients with moderate ascites. ^c^ Extrahepatic metastases include metastasis to one or more sites such as the lung, bone, and peritoneum.

## Result

### Patient

A total of 288 patients were included in this study, with a median age of 56 years (range, 25–80 years), of whom 255 (88.5%) were male. Baseline characteristics were comparable between the Icaritin–TACE group and the TACE alone group. Hepatitis B virus infection was the predominant underlying etiology in both groups (95.8% vs. 94.4%). Type II portal vein tumor thrombus was observed in 20.1% and 29.9% of patients, respectively. The mean serum albumin levels were 36.2 ± 4.8 g/L in the Icaritin–TACE group and 35.3 ± 4.6 g/L in the TACE alone group. Median serum AFP levels were 163.6 ng/mL and 58.9 ng/mL, respectively. Additionally, 50.0% of patients in the Icaritin–TACE group and 44.4% in the TACE alone group underwent three or more TACE sessions. The median maximum lesion diameter was 6.2 cm (IQR, 6.7) in the Icaritin–TACE group, with 62.5% of patients having lesions ≥5 cm, compared to 6.4 cm (IQR, 6.5) and 56.9% in the TACE alone group. None of these baseline variables showed statistically significant differences between the two groups (all P > 0.05; **Table 1**). In addition, temporary treatment interruption occurred in 86.8% of patients, primarily due to additional interventional procedures, targeted therapy, or immunotherapy; the duration of these interruptions ranged from approximately 1 week to 1 month, after which therapy was resumed. Dose reduction (to 300 mg twice daily) was implemented in 5.6% of patients who continued treatment thereafter, while 8.3% permanently discontinued Icaritin due to adverse events.

### OS and PFS

**Figure 2:**
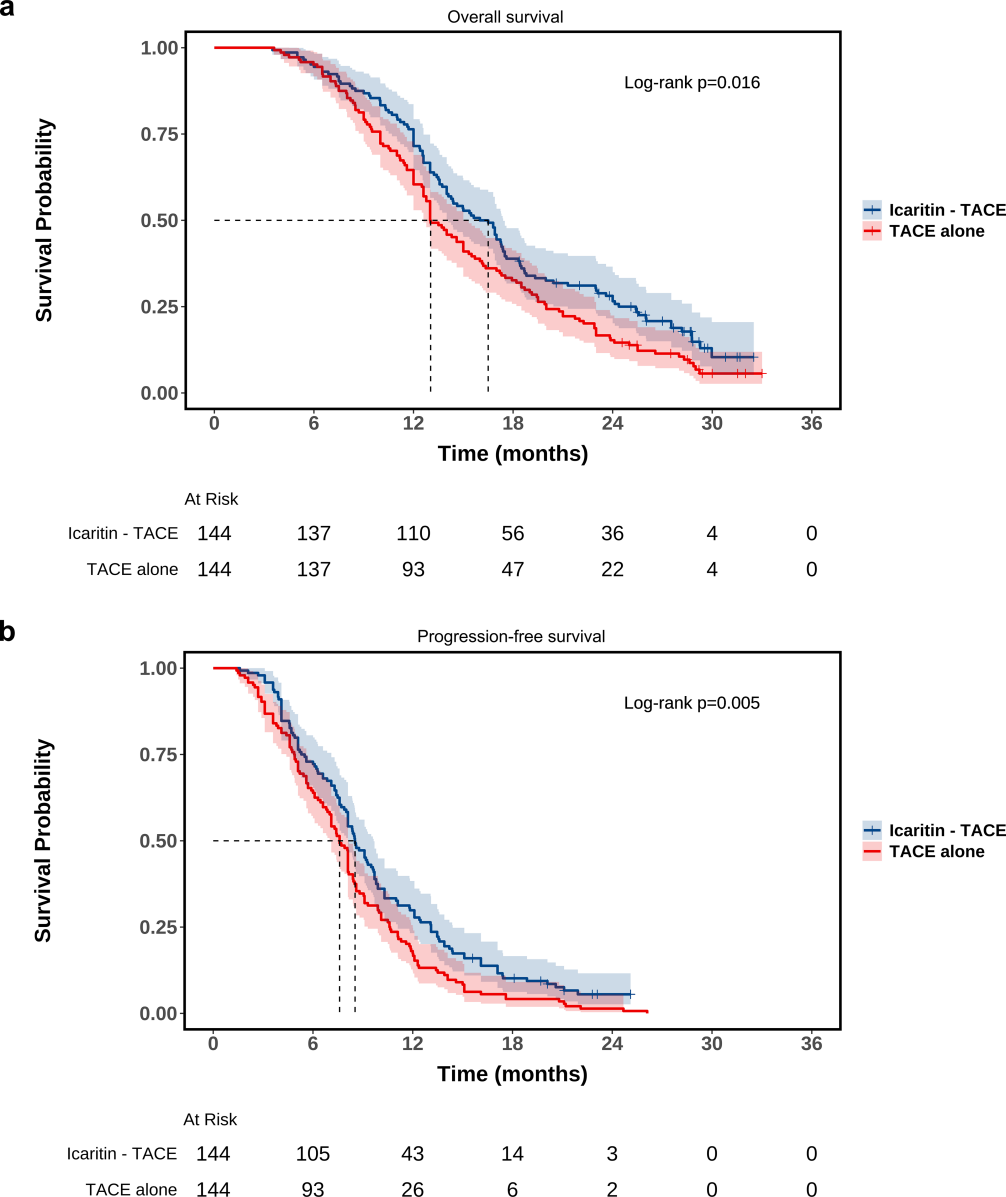
Kaplan–Meier curves for OS and PFS in the Icaritin–TACE and TACE-alone groups. **(A)** The Icaritin–TACE group showed 1-year and 2-year OS rates of 71.5% and 28.1%, respectively, compared to 60.5% and 15.3% in the TACE-alone group. **(B)** In the Icaritin–TACE cohort, 1-year and 2-year PFS rates were 29.9% and 5.5%, respectively, compared to 18.1% and 1.4% in the TACE-alone group. OS, overall survival; PFS, progression-free survival; TACE, transarterial chemoembolization.

The median follow-up duration was 14.5 months (range, 3.5–33.0 months). There were 119 deaths (82.6%) in the Icaritin-TACE group and 133 (92.4%) in the TACE alone group. Median OS was longer in the Icaritin–TACE group than in the TACE group (16.3 vs. 13.3 months; HR = 0.74, 95% CI: 0.58–0.94; P = 0.016) (**Figure 2A**). Cox proportional hazards modeling identified ECOG performance status (HR = 1.43, 95% CI: 1.04–1.97; P = 0.002), AFP level (HR = 0.71, 95% CI: 0.53–0.94; P = 0.017), number of tumor lesions (HR = 1.57, 95% CI: 1.14–2.16; P = 0.005), and maximum tumor diameter (HR = 0.66, 95% CI: 0.50–0.87; P = 0.004) as independent prognostic indicators for OS (**Supplementary Table 1**).

A total of 278 patients developed disease progression during follow-up, comprising 134 individuals (93.1%) in the Icaritin–TACE group and all 144 patients (100.0%) in the TACE monotherapy group. Median PFS was significantly longer in the Icaritin–TACE cohort compared to TACE alone (8.5 vs. 7.6 months; HR = 0.71, 95% CI: 0.56–0.91; P = 0.005) (**Figure 2B**). Furthermore, both univariate and multivariate Cox regression identified the number of tumor lesions as an independent prognostic factor for PFS (HR = 1.34; 95% CI: 1.01–1.78; P = 0.041) (**Supplementary Table 2**).

### Subgroup analyses

**Figure 3:**
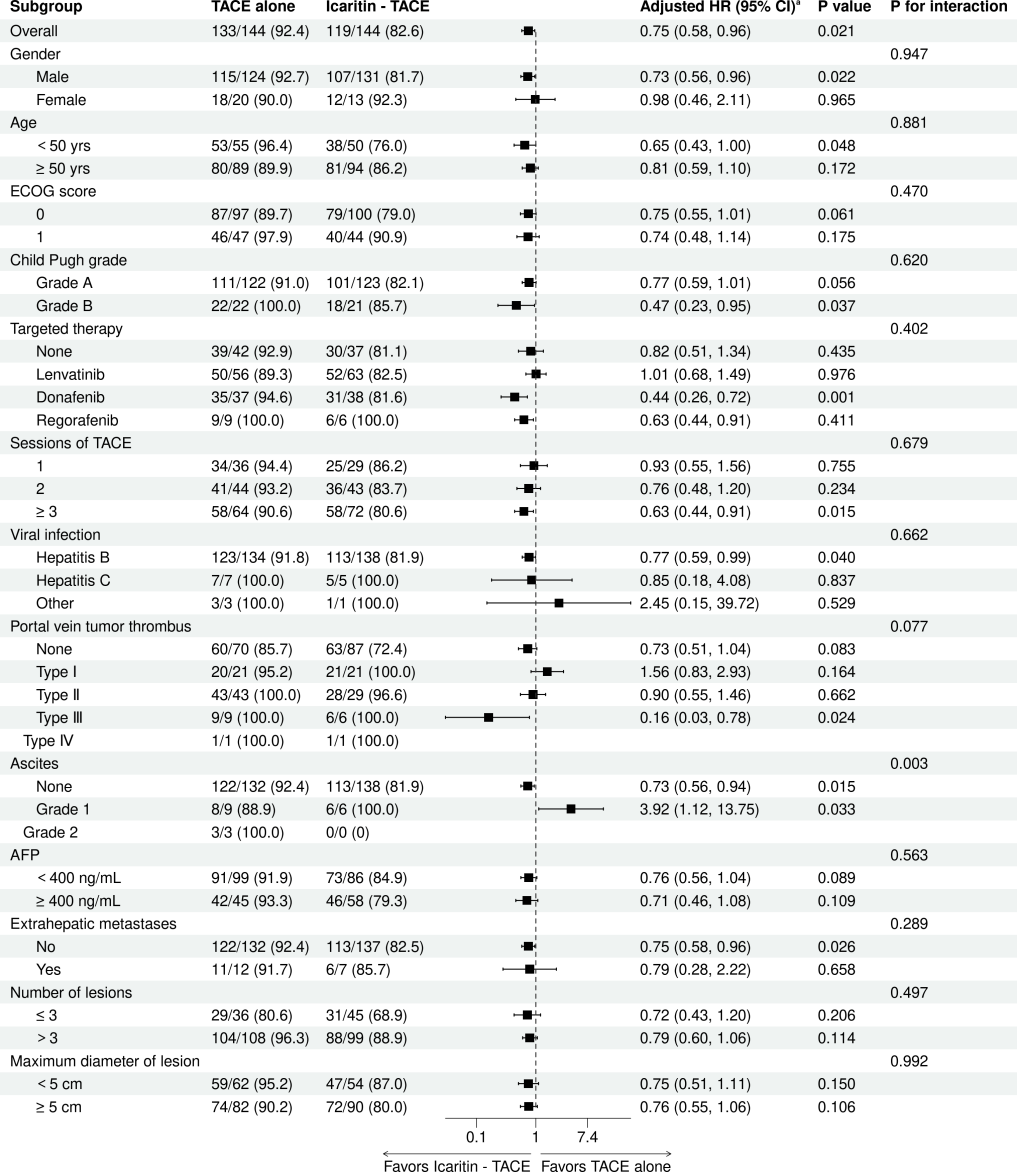
Subgroup analysis of OS stratified by treatment arm. ^a^, adjusted for Gender, Age, ECOG score, Child Pugh grade, Targeted therapy, Sessions of TACE, AFP, Extrahepatic metastases, Number of lesions, and Maximum diameter of lesion. Cox regression estimated HRs with 95% CIs across predefined subgroups. Icaritin–TACE significantly improved OS in patients receiving Donafenib (HR = 0.44), with HBV infection (HR = 0.77), without ascites (HR = 0.73), or without extrahepatic metastasis (HR = 0.75) (all P < 0.05). In contrast, grade 1 ascites was associated with increased mortality (HR = 3.92). Arrows indicate CIs exceeding axis limits. OS, overall survival; TACE, transarterial chemoembolization; HR, hazard ratio; CI, confidence interval.

Subgroup analyses using forest plots indicated that the combination treatment offered consistent advantages in both OS and PFS across most predefined categories—including male sex, patients receiving three or more TACE procedures, and those without ascites or with only mild ascites (grade 1) (**Figure 3**; **Supplementary Figure 1**).

Predefined subgroup analyses using the Cox model showed that Icaritin–TACE was associated with a lower risk of mortality among male patients (HR = 0.73, 95% CI: 0.56–0.96; P = 0.022). Comparable trends were observed in patients < 50 years (HR = 0.65, 95% CI: 0.43–1.00; P = 0.048), those with Child–Pugh class B liver function (HR = 0.47, 95% CI: 0.23–0.95; P = 0.037), and individuals who underwent ≥ 3 TACE sessions (HR = 0.63, 95% CI: 0.44–0.91; P = 0.015). Due to limited sample size, subgroup analyses for patients with type IV PVTT and grade 2 ascites were not conducted (**Figure 3**).

More specifically, Icaritin–TACE was associated with a significantly reduced risk of disease progression in several subgroups: patients aged ≥50 years (HR = 0.70; 95% CI: 0.52–0.94; P = 0.017), those with ECOG performance status of 1 (HR = 0.74; 95% CI: 0.55–0.98; P = 0.038), individuals with Child–Pugh class A liver function (HR = 0.73; 95% CI: 0.56–0.94; P = 0.017), and those with baseline AFP < 400 ng/mL (HR = 0.67; 95% CI: 0.49–0.90; P = 0.008). Additionally, interaction tests revealed statistically significant subgroup-by-treatment interactions for several variables, indicating that the treatment effect varied across these subgroups (**Figure 3**; **Supplementary Figure 1**). Due to limited sample size, subgroups involving non-HBV viral etiologies, type IV PVTT, and grade 2 ascites were excluded from the analysis. There were no statistically significant differences between groups in either the number of sequential TACE sessions or the frequency of imaging assessments (P > 0.05). Formal testing of the proportional hazards assumption using Schoenfeld residuals indicated no significant violations for either OS or PFS models (global PH test P > 0.10), supporting the validity of the Cox regression analyses.

### Tumor response

**Figure 4:**
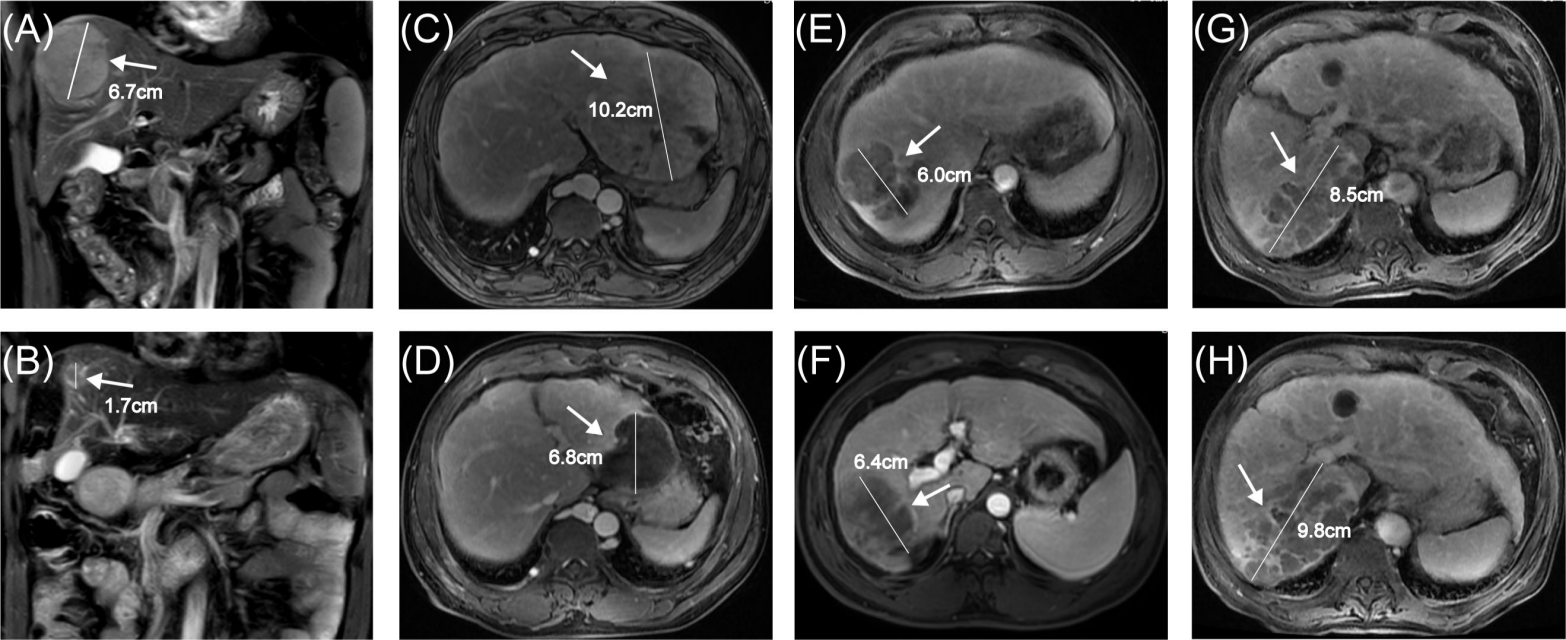
Tumor responses on CE-MRI in patients with advanced HCC and MVI treated with Icaritin plus TACE. Patient 1 **(A, B)**: A 53-year-old male with a 20-year HBV history. Baseline CE-MRI revealed a lesion in the superior right hepatic lobe (6.7 cm; A). After six cycles of Icaritin + TACE, the lesion regressed to 1.7 cm with no viable tumor at month 16. Response: complete response **(B)**. Patient 2 **(C, D)**: A 52-year-old male with HCC. Initial MRI showed multiple lesions in the left lobe, the largest measuring 10.2 cm with intense arterial enhancement **(C)**. After five cycles, MRI at month 15 showed shrinkage to 6.8 cm with partial enhancement. Response: partial response **(D)**. Patient 3 **(E, F)**: A 47-year-old male with >10-year HBV history. Baseline MRI showed a 6.0 cm lesion in the right posterior lobe and tumor thrombus in the IVC and hepatic vein **(E)**. After five cycles, enhancement and thrombus persisted. Response: stable disease **(F)**. Patient 4 **(G, H)**: A 60-year-old male with a 40-year HBV history. Pre-TACE MRI showed multiple hepatic lesions, including an 8.5 cm dominant mass **(G)**. After five cycles, the lesion enlarged to 9.8 cm. Response: progressive disease **(H)**. CE-MRI, contrast-enhanced magnetic resonance imaging; HCC, hepatocellular carcinoma; MVI, macrovascular invasion; TACE, transarterial chemoembolization; HBV, hepatitis B virus.

The inter-reader agreement for mRECIST categorization was substantial (Cohen’s κ = 0.82), confirming high consistency between radiologists. According to the mRECIST, there were no statistically significant differences between the Icaritin–TACE and TACE monotherapy groups in terms of complete response, partial response, or stable disease rates (all P > 0.05; **Table 2**). **Figure 4** presents representative tumor response outcomes from patients treated with Icaritin–TACE. However, the incidence of progressive disease was significantly lower in the Icaritin–TACE group (16.0% vs. 27.8%; P = 0.004). Although the ORR was comparable between groups (53.5% vs. 46.5%; P = 0.239), the DCR was higher among patients receiving Icaritin–TACE (84.0% vs. 72.2%; P = 0.015). Of particular interest, one patient in the Icaritin–TACE cohort achieved a durable complete response lasting more than 27.7 months.

**Table 2:** Best tumor response following treatment with icaritin–TACE versus TACE alone.

Response Category	Icaritin-TACE group (n = 144)	TACE group (n = 144)	*P*
Complete response	16 (11.1%)	11 (7.6%)	0.312
Partial response	61 (42.4%)	56 (38.9%)	0.549
Stable disease	44 (30.6%)	37 (25.7%)	0.293
Progressive disease	23 (16.0%)	40 (27.8%)	0.004
Objective response rate	77 (53.5%)	67 (46.5%)	0.239
Disease control rate	121 (84.0%)	104 (72.2%)	0.015

Icaritin -TACE, transarterial chemoembolization plus Icaritin; TACE, transarterial chemoembolization. The objective response rate was defined as the proportion of patients with complete response plus partial response. The disease control rate was defined as the proportion of patients with complete response plus partial response and stable disease. P values calculated by Chi-square test.

### Sensitivity analysis

The Love plot for the PSM cohort is shown in **Supplementary Figure 2**. **Supplementary Tables 3**, **4** summarize the baseline characteristics of patients treated with Icaritin–TACE versus TACE alone before and after PSM. After matching, 121 patients remained in each group. As shown in **Supplementary Table 3**, baseline covariates were well balanced between the two groups after PSM (all P > 0.05, before and after matching). Cox proportional hazards analyses performed on the matched cohort demonstrated risk estimates similar to those observed in the unmatched population. Based on the matched data, OS, PFS, and tumor response were reanalyzed. The median OS was 16.0 months in the Icaritin–TACE group and 13.0 months in the TACE-alone group (HR = 0.75, 95% CI: 0.57–0.98; P = 0.037), while the median PFS was 8.6 months and 7.6 months, respectively (HR = 0.69, 95% CI: 0.54–0.90; P = 0.005) (**Supplementary Figure 3**). Tumor response rates were comparable between the two groups (**Supplementary Table 5**). Collectively, these PSM-based sensitivity analyses support the robustness of our primary findings.

**Figure 5:**
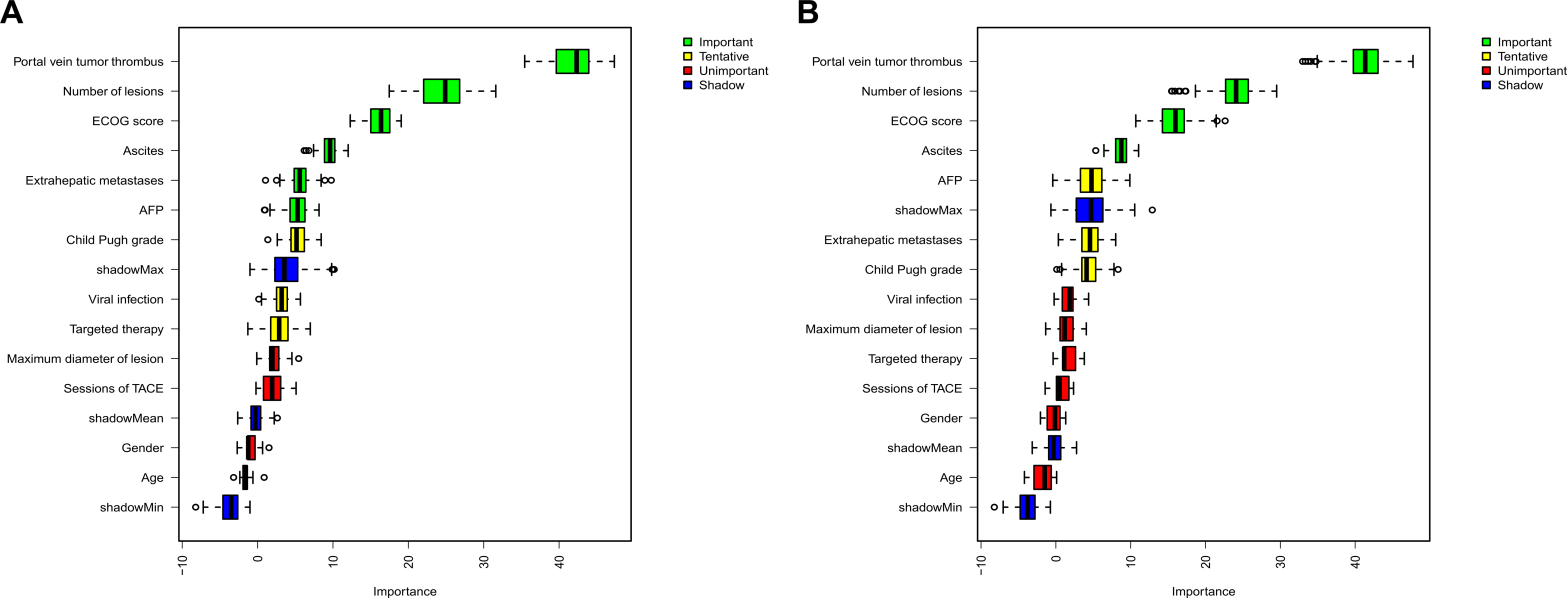
The Boruta algorithm was used to screen and rank the importance of clinical variables related to overall survival (OS). **(A)** Data before propensity score matching (PSM). **(B)** Data after PSM. X-axis (Importance): It shows the importance score of this variable. Y-axis (Variables): It lists all the clinical variables involved in the screening. Color: Green (Confirmed): Indicates that the algorithm confirms that this variable is important. Red (Rejected): Indicates that the algorithm considers this variable unimportant. Yellow (Tentative): indicates that the algorithm is uncertain.

**Figure 6:**
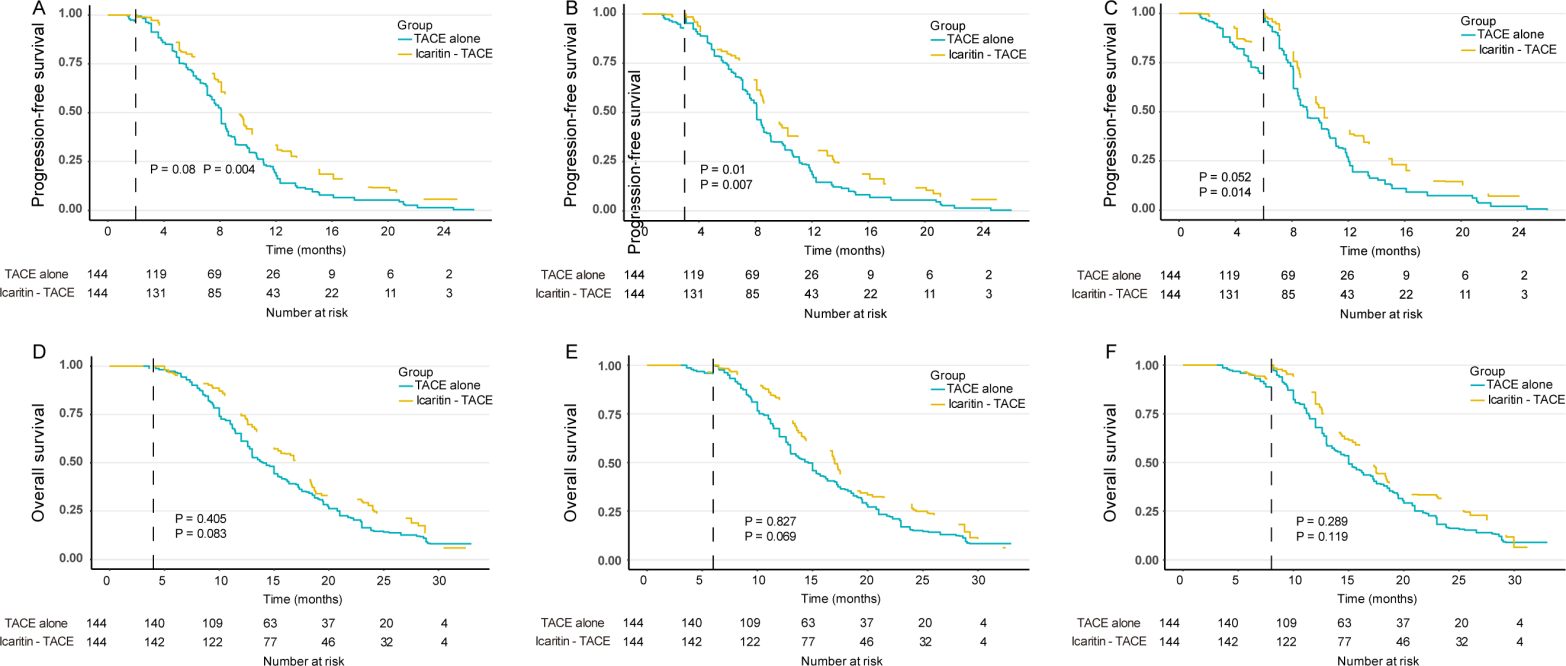
Landmark survival analysis for evaluating the impact of early events on OS and PFS in the Icaritin–TACE and TACE-alone groups. **(A)** PFS analysis with a 2-month cut-off point; **(B)** PFS analysis with a 3-month cut-off point; **(C)** PFS analysis with a cut-off point of 6 months; **(D)** OS analysis with a cut-off point of 4 months; **(E)** OS analysis with a 6-month cut-off point; **(F)** OS analysis with an 8-month cut-off point.

Using the Boruta algorithm, variables were classified as Important, Tentative, or Unimportant for predicting OS and PFS (**Figure 5**; **Supplementary Figure 4**). Variables identified as Unimportant were excluded from the primary multivariable Cox models (**Supplementary Tables 6**, **7**). Maximum lesion diameter, although labeled Tentative by Boruta, was retained due to its statistical significance (P < 0.05) and clinical relevance. A full multivariable model including all covariates significant in univariate analyses yielded consistent effect estimates and conclusions, confirming the robustness of the findings. No substantial multicollinearity was observed (all variance inflation factors < 5) (**Supplementary Table 8**). Landmark sensitivity analyses (PFS landmarks at 2, 3, and 6 months; OS landmarks at 4, 6, and 8 months) demonstrated results consistent with the primary Cox models, indicating that the observed survival benefit of Icaritin–TACE was robust to immortal-time and surveillance-frequency biases (**Figure 6**).

### Adverse events

AEs were graded in accordance with the National Cancer Institute Common Terminology Criteria for Adverse Events (CTCAE), version 4.03, and are comprehensively listed in **Supplementary Table 9**. No treatment-related deaths or unexpected toxicities were observed during the study period. All AEs were effectively managed with symptomatic or supportive interventions. Typical post-embolization symptoms—including abdominal discomfort, elevations in total bilirubin, ALT, AST, and GGT, as well as nausea, vomiting, and fever—were observed at similar frequencies in both the Icaritin–TACE and TACE monotherapy cohorts (all P > 0.05). Diarrhea, albuminuria, and hypertension were more frequently reported in the Icaritin–TACE group compared to TACE alone (all P < 0.05); however, these AEs were predominantly mild to moderate (Grade 1–2) and resolved rapidly following appropriate management. There were no statistically significant differences between the two groups in the occurrence of Grade 3–4 AEs (all P > 0.05). Dose adjustments of Icaritin were required in 13 patients (9.0%), while permanent discontinuation due to drug intolerance was necessary in 2 patients (1.4%).

## Discussion

In this multicenter retrospective analysis involving Chinese patients with advanced HCC and MVI, the combination of Icaritin with TACE was associated with improved clinical outcomes compared to TACE alone. Patients in the Icaritin–TACE group exhibited significantly longer median OS and PFS, as well as a higher DCR. These findings are particularly relevant given the poor prognosis typically observed in HCC patients with vascular involvement. The survival advantage observed in our study aligns with previous evidence from trials evaluating TACE combined with systemic therapies. For instance, a randomized phase III study in patients with recurrent intermediate-stage HCC and microvascular invasion demonstrated that TACE combined with sorafenib significantly prolonged OS (22.2 vs. 15.1 months) and PFS (16.2 vs. 11.8 months) compared with TACE alone (18). Likewise, the LAUNCH trial reported superior outcomes for lenvatinib plus TACE versus lenvatinib monotherapy in advanced-stage HCC, with OS and PFS extended to 17.8 and 10.6 months, respectively, compared to 11.5 and 6.4 months (5). Our findings broaden the application of combination regimens by incorporating a traditional Chinese medicine–derived immunomodulator, supporting Icaritin–TACE as a promising therapeutic strategy for patients with MVI-positive HCC.

Early preclinical investigations revealed that Icaritin promotes mitochondrial autophagy and apoptosis, thereby initiating ICD, facilitating tumor antigen release, and enhancing dendritic cell activation (12). When combined with TACE—which induces localized tumor necrosis—this ICD-enhancing effect may potentiate a stronger antitumor immune response (19). Mechanistically, Icaritin inhibits the IL-6/JAK/STAT3 pathway by targeting the MyD88/IкB complex, thereby reducing the production of pro-inflammatory cytokines such as IL-6 and TNF-α and downregulating PD-L1 expression within the tumor microenvironment, a pathway implicated in immune evasion in HCC (11, 20). Moreover, Icaritin suppresses tumor-associated extramedullary hematopoiesis in the spleen, thereby limiting the expansion and activity of immunosuppressive myeloid-derived suppressor cells (MDSCs) and promoting T cell–mediated cytotoxicity (21). These immunomodulatory properties—ICD induction, cytokine suppression, and MDSC inhibition—are likely responsible for the enhanced tumor control observed with the Icaritin–TACE combination. The therapeutic rationale for combining Icaritin with TACE is thus grounded in complementary mechanisms that address both local and systemic disease processes. TACE achieves ischemic necrosis through selective arterial embolization but induces post-embolization hypoxia that activates proangiogenic and immunosuppressive signaling, potentially facilitating tumor recurrence. Icaritin, by contrast, enhances T cell–mediated cytotoxicity and counteracts TACE-induced immunosuppression (21). This complementary interaction suggests a theoretically synergistic relationship: TACE reduces tumor bulk and releases tumor-associated antigens, whereas Icaritin amplifies systemic antitumor immunity and mitigates pro-tumor inflammatory signaling. The consistent improvement in OS, PFS, and DCR observed in this study supports that this biological synergy may translate into clinically meaningful benefit.

As one of the first plant-based flavonoid immunotherapeutics in oncology, Icaritin offers a mechanism distinct from that of tyrosine kinase inhibitors and checkpoint blockade agents. Its approval in China reflects both its demonstrated clinical benefit and manageable safety profile. Clinically, Icaritin has been employed in various combination regimens, particularly in hepatitis B virus–related or unresectable HCC cases (13). Our study further highlights Icaritin’s regulatory and clinical significance: by integrating an approved immunomodulator with TACE, we harness a synergy between locoregional tumor kill and systemic immune modulation. The OS benefit aligns with the phase III trial (13.54 months in CBS+ subgroup) but extends it to MVI populations—previously excluded from pivotal studies (14). Our lower median OS reflects the adverse prognosis of MVI, validating real-world applicability.

Within the BCLC staging system, HCC accompanied by MVI is categorized as stage C, indicating advanced disease. Although Western guidelines—such as those from the European Association for the Study of the Liver (EASL) and the American Association for the Study of Liver Diseases (AASLD)—typically recommend systemic therapy rather than TACE for BCLC-C patients, a subset with PVTT may retain preserved hepatic function and remain clinically asymptomatic (22, 23). Prognosis in these individuals is strongly influenced by the extent of PVTT, particularly in the presence of elevated AFP levels or substantial tumor burden. Curative resection is often contraindicated due to occult hepatic synthetic impairment and the risk of impending decompensation. Nevertheless, patients with limited PVTT involvement, such as those classified as PV1 (thrombus restricted to first-order branches), may still benefit from surgical or interventional therapies (22). By contrast, both the Pan-Asian adaptation of the ESMO Clinical Practice Guidelines and the China Liver Cancer Staging framework favor a more intervention-oriented treatment approach (24, 25). These recommendations support the use of TACE and other locoregional modalities, even for patients with advanced HCC, particularly when combined with systemic agents such as targeted therapies or ICIs. Notably, expert consensus in the Guidelines for the Diagnosis and Treatment of Primary Liver Cancer issued by leading Chinese authorities emphasizes that combining systemic and locoregional modalities may improve objective response rates and increase the potential for conversion to resectable disease (16). Our results support this therapeutic approach. The survival benefit of combining Icaritin with TACE may stem from the complementary effects of local tumor control and systemic immune modulation. Additionally, Icaritin may enhance HBP1-mediated repression of AFP, thereby weakening AFP’s influence on PTEN, MMP9, and caspase-3, which could inhibit tumor proliferation and migration while promoting apoptosis (26).

When compared with other combination regimens, Icaritin–TACE may offer distinct advantages. Unlike tyrosine kinase inhibitors or anti-VEGF antibodies, Icaritin demonstrates a more favorable safety profile, which could be particularly valuable in patients who are unsuitable for cytotoxic or highly immunogenic therapies (5, 18, 27, 28). Although diarrhea was more frequently observed in the Icaritin–TACE group than in the TACE-alone cohort, it was generally mild to moderate (Grade 1–2) and manageable with supportive care, without resulting in treatment discontinuation in most cases. In addition, mild elevations in albuminuria and hypertension were also noted, likely reflecting Icaritin’s immunomodulatory and endothelial effects rather than direct organ toxicity. These events were reversible and did not necessitate permanent treatment cessation. Importantly, unlike intravenous immune checkpoint inhibitors—which are associated with immune-related adverse events and logistical barriers—Icaritin is orally administered and has demonstrated good tolerability, even in patients with cirrhosis. It is important to acknowledge that Icaritin’s clinical use and regulatory approval are currently limited to China. Its therapeutic role in broader international populations remains to be clarified through future clinical trials and global regulatory evaluations.

This study has several limitations. Its retrospective design may introduce selection and outcome assessment biases. Because Icaritin was preferentially used in a biomarker-informed clinical context, whereas the comparator arm consisted of unselected TACE recipients, residual selection bias related to CBS status cannot be fully excluded. Although baseline characteristics were balanced, unmeasured confounders such as tumor biology or subsequent therapies may affect results. The cohort mainly included Chinese HBV-related HCC patients, limiting generalizability to other etiologies or populations. Certain high-risk subgroups, such as those with Cheng’s type IV PVTT or grade 2 ascites, were underrepresented. Thus, the benefit of Icaritin in these groups remains unclear. As a cohort study, causality cannot be confirmed; prospective randomized trials are needed to validate findings and optimize combination strategies involving Icaritin.

## Conclusion

In summary, for advanced HCC patients with MVI, the combination of Icaritin and TACE improves disease control and survival outcomes compared to TACE alone. These findings highlight the potential value of integrating an oral immunomodulator with locoregional therapy in high-risk populations and warrant confirmation in future prospective studies.

## Data Availability

The raw data supporting the conclusions of this article will be made available by the authors, without undue reservation.
