# Supplementary figures and images for: Icaritin plus TACE improves survival in advanced HCC with macrovascular invasion: a multicenter cohort study
Source: Front Immunol. 2026 May 29;17:1684486. doi: 10.3389/fimmu.2026.1684486 (PMC13260649; doi:10.3389/fimmu.2026.1684486)

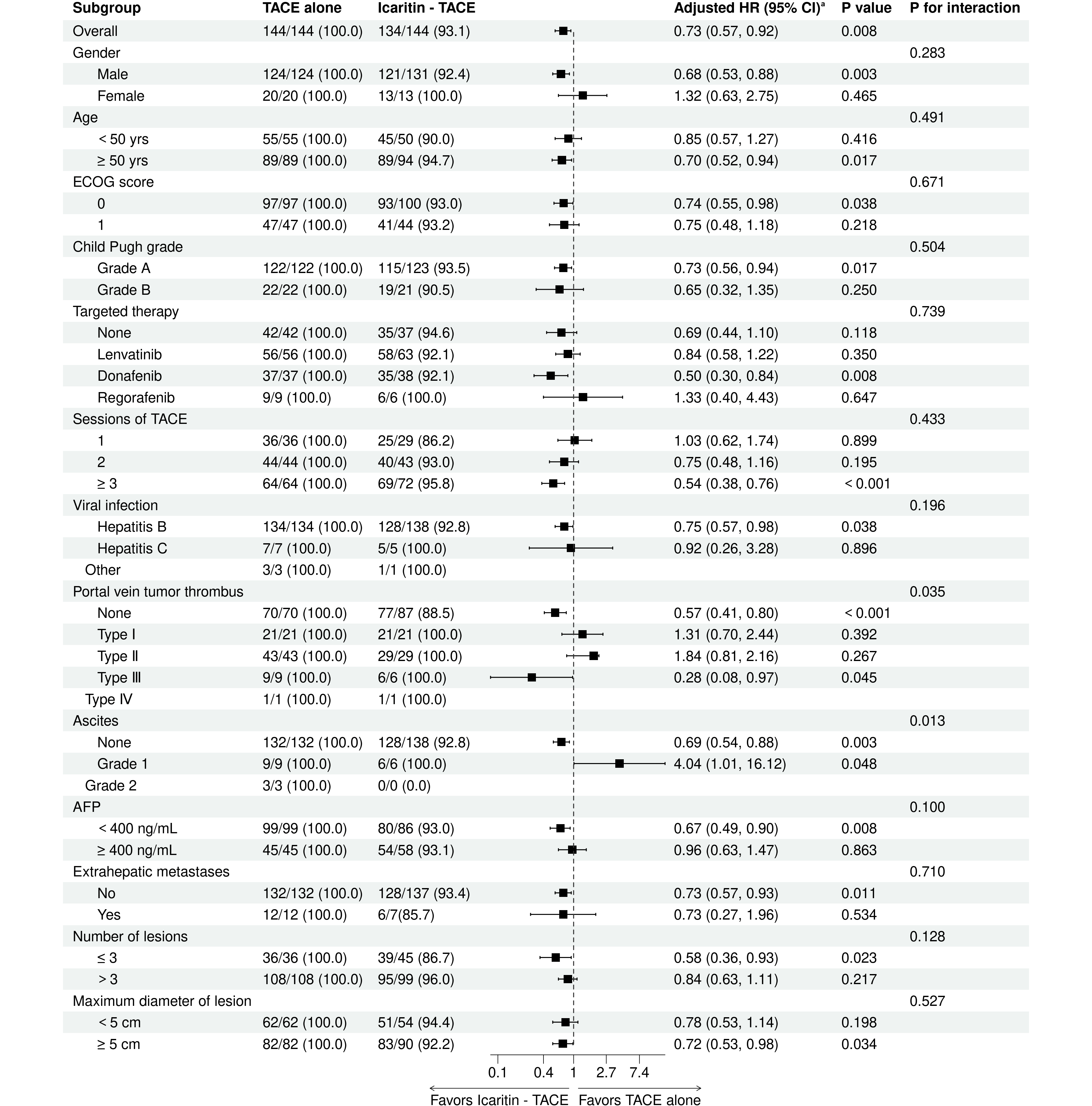

Supplement: Supplementary file 1 [file Image1.tiff]

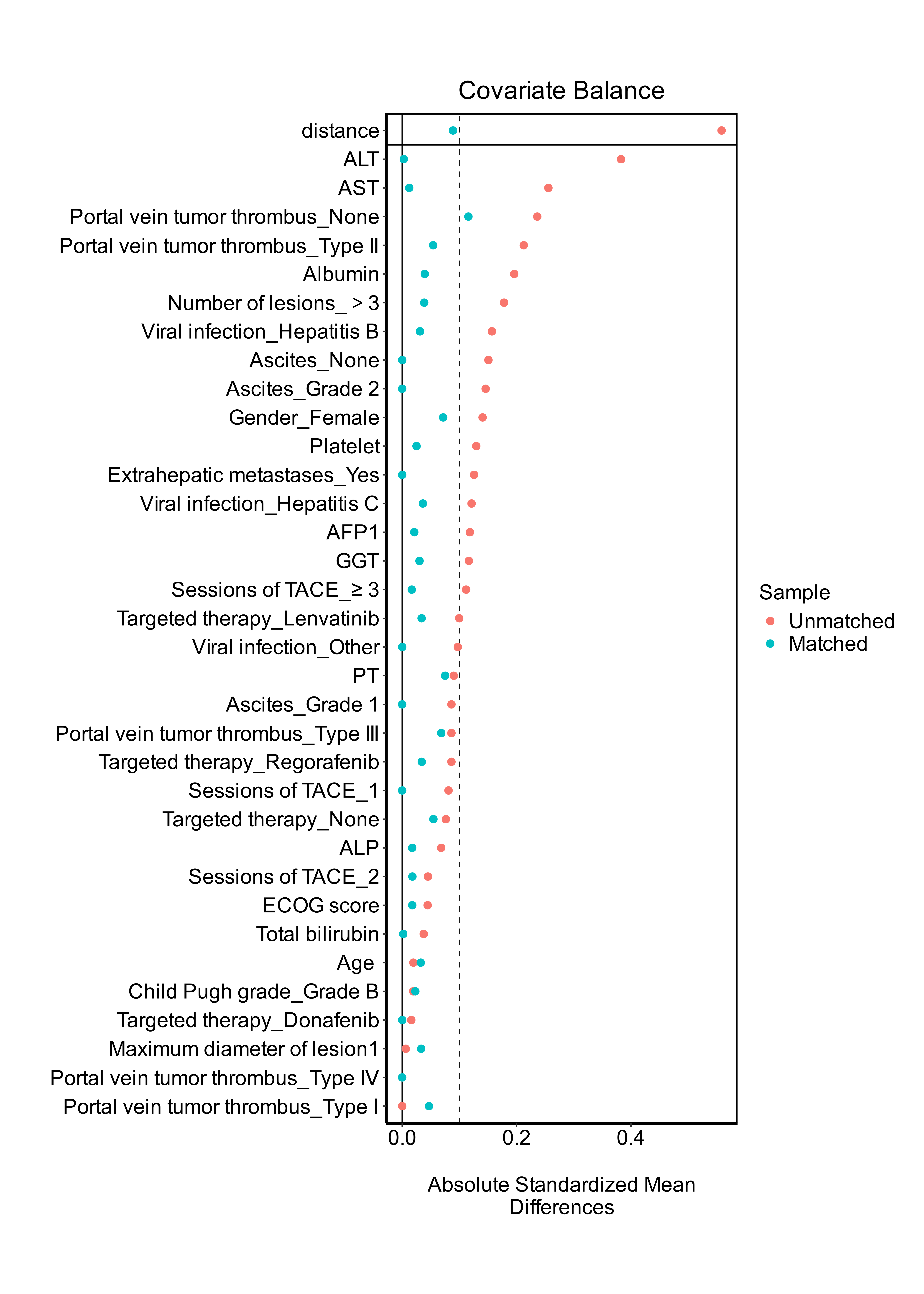

Supplement: Supplementary file 2 [file Image2.tiff]

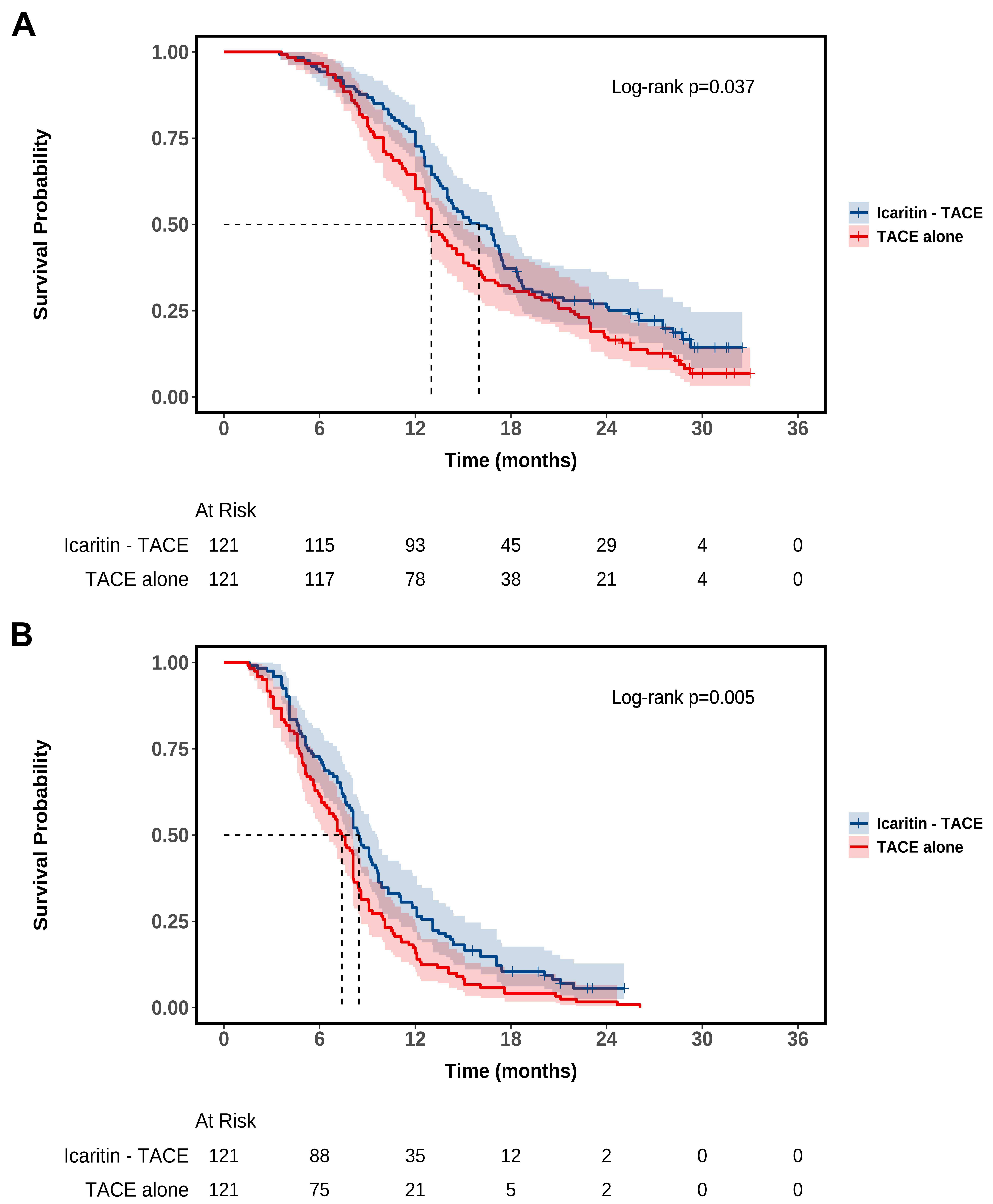

Supplement: Supplementary file 3 [file Image3.tiff]

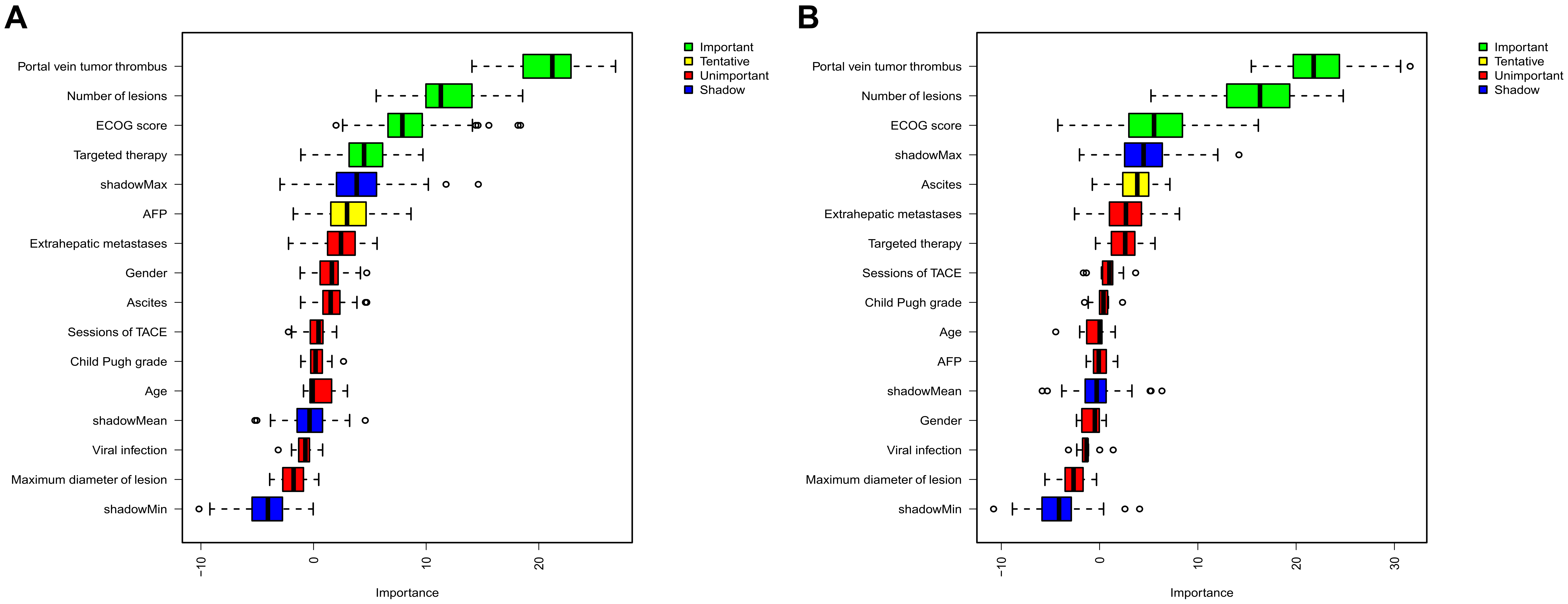

Supplement: Supplementary file 4 [file Image4.tiff]
