# Supplementary material for: Icaritin plus TACE improves survival in advanced HCC with macrovascular invasion: a multicenter cohort study
Source: Front Immunol. 2026 May 29;17:1684486. doi: 10.3389/fimmu.2026.1684486 (PMC13260649; doi:10.3389/fimmu.2026.1684486)
Supplement: Supplementary file 5 [file Table1.docx]

| **Supplementary Table 1. Univariate and Multivariate COX Analysis of Overall Survival after Treatment** | | | | | | | |
| --- | --- | --- | --- | --- | --- | --- | --- |
|  |  | Univariate | | | Multivariate | | |
|  |  | HR | 95%CI | *P* | HR | 95%CI | *P* |
| Gender (ref. Male) | | 1.21 | 0.83 ~ 1.77 | 0.328 |  |  |  |
| Age (ref.＜50yrs) | | 1.01 | 0.78 ~ 1.30 | 0.953 |  |  |  |
| ECOG score (ref. 0) | | 1.99 | 1.52 ~ 2.59 | <0.001 | 1.43 | 1.04 ~ 1.97 | 0.002 |
| Child-Pugh grade (ref. Grade A) | | 1.35 | 0.96 ~ 1.90 | 0.082 | 1.11 | 0.76 ~ 1.61 | 0.597 |
| Targeted therapy (ref. None) | | | |  |  |  |  |
| Lenvatinib | | 0.98 | 0.72 ~ 1.33 | 0.906 | 0.95 | 0.69 ~ 1.30 | 0.736 |
| Donafenib | | 1.27 | 0.91 ~ 1.78 | 0.163 | 1.28 | 0.90 ~ 1.84 | 0.174 |
| Regorafenib | | 1.87 | 1.07 ~ 3.28 | 0.029 | 1.77 | 0.97 ~ 3.22 | 0.064 |
| Sessions of TACE (ref. 1) | |  |  |  |  |  |  |
| 2 | | 0.98 | 0.70 ~ 1.38 | 0.905 |  |  |  |
| ≥3 | | 1.01 | 0.74 ~ 1.38 | 0.961 |  |  |  |
| \| Viral infection (ref. Hepatitis B) \|  \| \| --- \| --- \| | | | | | | | |
| Hepatitis C | | 1.43 | 0.80 ~ 2.55 | 0.230 | 1.49 | 0.80 ~ 2.78 | 0.205 |
| Other | | 5.15 | 1.87 ~ 14.15 | 0.001 | 2.21 | 0.62 ~ 7.87 | 0.222 |
| Portal vein tumor thrombus (ref. None)^a^ | |  |  |  |  |  |  |
| Type Ⅰ | | 2.01 | 1.40 ~ 2.88 | <0.001 | 1.93 | 1.31 ~ 2.84 | <0.001 |
| Type Ⅱ | | 3.08 | 2.27 ~ 4.17 | <0.001 | 2.68 | 1.92 ~ 3.74 | <0.001 |
| Type Ⅲ | | 5.98 | 3.42 ~ 10.45 | <0.001 | 5.52 | 2.87 ~ 10.63 | <0.001 |
| Type Ⅳ | | 2.28 | 0.56 ~ 9.27 | 0.251 | 2.78 | 0.65 ~ 11.89 | 0.169 |
| Ascites (ref. None)^b^ | |  |  |  |  |  |  |
| Grade 1 | | 2.20 | 1.28 ~ 3.78 | 0.004 | 1.09 | 0.58 ~ 2.06 | 0.794 |
| Grade 2 | | 5.64 | 1.76 ~ 18.02 | 0.004 | 0.77 | 0.16 ~ 3.58 | 0.734 |
| AFP (ref.＜400ng/mL) | | 0.78 | 0.60 ~ 1.01 | 0.064 | 0.71 | 0.53 ~ 0.94 | 0.017 |
| Extrahepatic metastases (ref. None) | | 1.75 | 1.07 ~ 2.88 | 0.026 | 0.98 | 0.56 ~ 1.71 | 0.954 |
| Number of lesions (ref. ≤3) | | 2.10 | 1.56 ~ 2.82 | <0.001 | 1.57 | 1.14 ~ 2.16 | 0.005 |
| Maximum diameter of lesion (ref.＜5cm) | | 0.74 | 0.57 ~ 0.95 | 0.020 | 0.66 | 0.50 ~ 0.87 | 0.004 |

Abbreviations: ECOG, Eastern Cooperative Oncology Group; AFP, Alpha-Fetoprotein. ^a^ According to the Cheng's classification (Cheng Shuqin classification) used in China. Type Ⅰ: the tumor thrombus is located in the portal vein branches of the hepatic segment or subsegment; Type Ⅱ: tumor thrombus invades the left or right branch of the portal vein; Type Ⅲ: tumor thrombus involves the main trunk of the portal vein; Type Ⅳ: tumor thrombus extends into the superior mesenteric vein or splenic vein. ^b^ Grade 1 indicates patients with mild ascites; Grade 2 indicates patients with moderate ascites.
