# Supplementary material for: Icaritin plus TACE improves survival in advanced HCC with macrovascular invasion: a multicenter cohort study
Source: Front Immunol. 2026 May 29;17:1684486. doi: 10.3389/fimmu.2026.1684486 (PMC13260649; doi:10.3389/fimmu.2026.1684486)
Supplement: Supplementary file 6 [file Table2.docx]

| **Supplementary Table 2. Univariate and Multivariate COX Analysis of Progression-free Survival after Treatment** | | | | | | | |
| --- | --- | --- | --- | --- | --- | --- | --- |
|  |  | Univariate | | | Multivariate | | |
|  |  | HR | 95%CI | *P* | HR | 95%CI | *P* |
| Gender (ref. Male) | | 0.88 | 0.61 ~ 1.26 | 0.486 |  |  |  |
| Age (ref.＜50yrs) | | 0.86 | 0.67 ~ 1.10 | 0.219 |  |  |  |
| ECOG score (ref. 0) | | 1.52 | 1.18 ~ 1.96 | <0.001 | 1.32 | 0.99 ~ 1.76 | 0.058 |
| Child-Pugh grade (ref. Grade A) | | 1.10 | 0.79 ~ 1.53 | 0.577 |  |  |  |
| Targeted therapy (ref. None) | |  |  |  |  |  |  |
| Lenvatinib | | 1.04 | 0.77 ~ 1.38 | 0.814 | 0.99 | 0.74 ~ 1.33 | 0.945 |
| Donafenib | | 1.33 | 0.96 ~ 1.83 | 0.087 | 1.32 | 0.95 ~ 1.84 | 0.102 |
| Regorafenib | | 1.85 | 1.06 ~ 3.23 | 0.031 | 1.64 | 0.91 ~ 2.94 | 0.098 |
| Sessions of TACE (ref. 1) | |  |  |  |  |  |  |
| 2 | | 1.16 | 0.83 ~ 1.61 | 0.388 |  |  |  |
| ≥3 | | 1.14 | 0.84 ~ 1.54 | 0.415 |  |  |  |
| \| Viral infection (ref. Hepatitis B) \|  \| \| --- \| --- \| | | | | | | | |
| Hepatitis C | | 1.29 | 0.72 ~ 2.31 | 0.384 |  |  |  |
| Other | | 1.61 | 0.60 ~ 4.33 | 0.347 |  |  |  |
| Portal vein tumor thrombus (ref. None)^a^ | |  |  |  |  |  |  |
| Type Ⅰ | | 1.65 | 1.17 ~ 2.33 | 0.005 | 1.53 | 1.07 ~ 2.19 | 0.020 |
| Type Ⅱ | | 1.79 | 1.34 ~ 2.38 | <0.001 | 1.52 | 1.12 ~ 2.06 | 0.007 |
| Type Ⅲ | | 2.83 | 1.65 ~ 4.87 | <0.001 | 2.89 | 1.58 ~ 5.28 | <0.001 |
| Type Ⅳ | | 1.83 | 0.45 ~ 7.42 | 0.398 | 1.61 | 0.38 ~ 6.92 | 0.519 |
| Ascites (ref. None)^b^ | |  |  |  |  |  |  |
| Grade 1 | | 1.64 | 0.97 ~ 2.77 | 0.063 | 0.98 | 0.54 ~ 1.76 | 0.941 |
| Grade 2 | | 1.76 | 0.56 ~ 5.51 | 0.335 | 0.63 | 0.18 ~ 2.23 | 0.474 |
| AFP (ref.＜400ng/mL) | | 0.83 | 0.65 ~ 1.07 | 0.149 |  |  |  |
| Extrahepatic metastases (ref. None) | | 1.37 | 0.85 ~ 2.22 | 0.198 |  |  |  |
| Number of lesions (ref. ≤3) | | 1.60 | 1.22 ~ 2.09 | <0.001 | 1.34 | 1.01 ~ 1.78 | 0.041 |
| Maximum diameter of lesion (ref.＜5cm) | | 0.91 | 0.71 ~ 1.16 | 0.428 |  |  |  |

Abbreviations: ECOG, Eastern Cooperative Oncology Group; AFP, Alpha-Fetoprotein. ^a^ According to the Cheng's classification (Cheng Shuqin classification) used in China. Type Ⅰ: the tumor thrombus is located in the portal vein branches of the hepatic segment or subsegment; Type Ⅱ: tumor thrombus invades the left or right branch of the portal vein; Type Ⅲ: tumor thrombus involves the main trunk of the portal vein; Type Ⅳ: tumor thrombus extends into the superior mesenteric vein or splenic vein. ^b^ Grade 1 indicates patients with mild ascites; Grade 2 indicates patients with moderate ascites.
