# Supplementary material for: Icaritin plus TACE improves survival in advanced HCC with macrovascular invasion: a multicenter cohort study
Source: Front Immunol. 2026 May 29;17:1684486. doi: 10.3389/fimmu.2026.1684486 (PMC13260649; doi:10.3389/fimmu.2026.1684486)
Supplement: Supplementary file 7 [file Table3.docx]

| Supplementary Table 3. Baseline covariates before and after matching | | | | | | | | |
| --- | --- | --- | --- | --- | --- | --- | --- | --- |
| Variables | Level | Before Matching | | |  | After Matching | | |
|  |  | Icaritin - TACE | TACE alone | SMD |  | Icaritin - TACE | TACE alone | SMD |
| n |  | 144 | 144 |  |  | 121 | 121 |  |
| Gender (%) | Male | 131 (91.0) | 124 (86.1) | -0.141 |  | 110 (90.9) | 107 (88.4) | -0.072 |
|  | Female | 13 (9.0) | 20 (13.9) | 0.141 |  | 11 (9.1) | 14 (11.6) | 0.072 |
| Age. (median (IQR)) |  | 55 (17.0) | 56 (17.0) | -0.020 |  | 56 (17.0) | 56 (17.0) | -0.032 |
| ECOG score (%)^a^ | 0 | 100 (69.4) | 97 (67.4) | -0.044 |  | 87 (71.9) | 86 (71.1) | -0.018 |
|  | 1 | 44 (30.6) | 47 (32.6) | 0.044 |  | 34 (28.1) | 35 (28.9) | 0.018 |
| Child Pugh grade (%) | Grade A | 123 (85.4) | 122 (84.7) | -0.019 |  | 105 (86.8) | 104 (86.0) | -0.023 |
|  | Grade B | 21 (14.6) | 22 (15.3) | 0.019 |  | 16 (13.2) | 17 (14.0) | 0.023 |
| Targeted therapy (%) | None | 37 (25.7) | 42 (29.2) | 0.076 |  | 33 (27.3) | 36 (29.8) | 0.055 |
|  | Lenvatinib | 63 (43.8) | 56 (38.9) | -0.100 |  | 51 (42.1) | 49 (40.5) | -0.034 |
|  | Donafenib | 38 (26.4) | 37 (25.7) | -0.016 |  | 31 (25.6) | 31 (25.6) | 0.000 |
|  | Regorafenib | 6 (4.2) | 9 (6.2) | 0.086 |  | 6 (5.0) | 5 (4.1) | -0.034 |
| Sessions of TACE (%) | 1 | 30 (20.8) | 35 (24.3) | 0.081 |  | 27 (22.3) | 27 (22.3) | 0.000 |
|  | 2 | 42 (29.2) | 45 (31.2) | 0.045 |  | 36 (29.8) | 37 (30.6) | 0.018 |
|  | ≥ 3 | 72 (50.0) | 64 (44.4) | -0.112 |  | 58 (47.9) | 57 (47.1) | -0.017 |
| Viral infection (%) | Hepatitis B | 139 (96.5) | 133 (92.4) | -0.157 |  | 116 (95.9) | 115 (95.0) | -0.031 |
|  | Hepatitis C | 4 (2.8) | 8 (5.6) | 0.121 |  | 4 (3.3) | 5 (4.1) | 0.036 |
|  | Other | 1 (0.7) | 3 (2.1) | 0.097 |  | 1 (0.8) | 1 (0.8) | 0.000 |
| Portal vein tumor thrombus (%) | None | 87 (60.4) | 70 (48.6) | -0.236 |  | 70 (57.9) | 63 (52.1) | -0.116 |
|  | Type Ⅰ | 21 (14.6) | 21 (14.6) | 0.000 |  | 17 (14.0) | 19 (15.7) | 0.047 |
|  | Type Ⅱ | 29 (20.1) | 43 (29.9) | 0.212 |  | 28 (23.1) | 31 (25.6) | 0.054 |
|  | Type Ⅲ | 6 (4.2) | 9 (6.2) | 0.086 |  | 5 (4.1) | 7 (5.8) | 0.068 |
|  | Type Ⅳ | 1 (0.7) | 1 (0.7) | 0.000 |  | 1 (0.8) | 1 (0.8) | 0.000 |
| Ascites (%)^b^ | None | 138 (95.8) | 132 (91.7) | -0.151 |  | 116 (95.9) | 116 (95.9) | 0.000 |
|  | Grade 1 | 6 (4.2) | 9 (6.2) | 0.086 |  | 5 (4.1) | 5 (4.1) | 0.000 |
|  | Grade 2 | 0 (0.0) | 3 (2.1) | 0.146 |  | 0 (0.0) | 0 (0.0) | 0.000 |
| Albumin (mean (SD)) |  | 36.2 (±4.8) | 35.3 (±4.6) | -0.196 |  | 35.9 (±4.7) | 35.8 (±4.6) | -0.040 |
| ALT (median (IQR)) |  | 54.0 (72.8) | 59.0 (65.8) | -0.383 |  | 50.9 (69.3) | 61.2 (71.6) | 0.003 |
| AST (median (IQR)) |  | 74.5 (95.0) | 60.5 (82.8) | -0.256 |  | 69.0 (77.4) | 59.3 (77.2) | -0.012 |
| ALP (median (IQR)) |  | 144,5 (129.3) | 167.0 (120.5) | 0.068 |  | 147.8 (141.2) | 153.0 (99.5) | 0.017 |
| GGT (median (IQR)) |  | 138.5 (165.0) | 131.0 (156.3) | -0.117 |  | 136.5 (156.4) | 130.0 (122.8) | -0.030 |
| Platelet (median (IQR)) |  | 127.0 (87.5) | 130.0 (105.3) | 0.130 |  | 129.1 (91.6) | 123.0 (96.0) | 0.025 |
| PT (median (IQR)) |  | 12.9 (1.9) | 13.1 (2.3) | 0.090 |  | 13.0 (2.0) | 13.0 (2.0) | 0.075 |
| Total bilirubin (median (IQR)) |  | 21.1 (13.7) | 20.2 (16.6) | 0.038 |  | 22.0 (11.2) | 20.3 (15.5) | 0.002 |
| AFP (median (IQR)) |  | 163.6 (990.7) | 58.9 (903.9) | -0.118 |  | 168.3 (990.3) | 43.1 (569.9) | 0.021 |
| Extrahepatic metastases (%)^c^ | No | 137 (95.1) | 132 (91.7) | -0.126 |  | 114 (94.2) | 114 (94.2) | 0.000 |
|  | Yes | 7 (4.9) | 12 (8.3) | 0.126 |  | 7 (5.8) | 7 (5.8) | 0.000 |
| Number of lesions (%) | ≤ 3 | 46 (31.9) | 35 (24.3) | -0.178 |  | 35 (28.9) | 33 (27.3) | -0.039 |
|  | ＞3 | 98 (68.1) | 109 (75.7) | 0.178 |  | 86 (71.1) | 88 (72.7) | 0.039 |
| Maximum diameter of lesion (median (IQR)) |  | 6.2 (6.7) | 6.4 (6.5) | -0.006 |  | 6.1 (5.8) | 6.1 (5.6) | -0.033 |
| Abbreviations: Icaritin -TACE, transarterial chemoembolization plus Icaritin; TACE, transarterial chemoembolization; SMD, Standardized Mean Difference ECOG, Eastern Cooperative Oncology Group; ALT, alanine aminotransferase; AST, aspartate aminotransferase; ALP, Alkaline Phosphatase; GGT, gamma-glutamyl transferase; PT, prothrombin time (international ratio); AFP, alpha-Fetoprotein. ^a^ ECOG score of 0 indicates that patient is fully active and able to carry on all pre-disease activities without restriction, and 1 indicates that patient is restricted in physically strenuous activity but is ambulatory and able to carry out work of a light nature, including self-care. ^b^ Grade 1 indicates patients with mild ascites; Grade 2 indicates patients with moderate ascites. ^c^ Extrahepatic metastases include metastasis to one or more sites such as the lung, bone, and peritoneum. | | | | | | | | |
