# Supplementary material for: Icaritin plus TACE improves survival in advanced HCC with macrovascular invasion: a multicenter cohort study
Source: Front Immunol. 2026 May 29;17:1684486. doi: 10.3389/fimmu.2026.1684486 (PMC13260649; doi:10.3389/fimmu.2026.1684486)
Supplement: Supplementary file 8 [file Table4.docx]

| **Supplementary Table 4. Comparison of Baseline Characteristics Between Patients Treated with Icaritin-TACE vs TACE Alone after Matching** | | | | | | |
| --- | --- | --- | --- | --- | --- | --- |
| **Characteristics** | **Unmatched** | | | **Matched** | | |
|  | **Icaritin – TACE group**   **(n = 144)^a^** | **TACE alone** **group  (n = 144)^a^** | ***P***^b^ | **Icaritin – TACE group**  **(n = 121)^a^** | **TACE alone** **group  (n = 121)^a^** | ***P***^b^ |
| **Gender** |  |  | 0.195 |  |  | 0.526 |
| Male | 131 (91.0%) | 124 (86.1%) |  | 110 (90.9%) | 107 (88.4%) |  |
| Female | 13 (9.0%) | 20 (13.9%) |  | 11 (9.1%) | 14 (11.6%) |  |
| **Age.** |  |  | 0.807 |  |  | 0.712 |
| Median (IQR) | 55 (17) | 56 (17) |  | 56 (17) | 56 (17) |  |
| **ECOG score^c^** |  |  | 0.704 |  |  | 0.887 |
| 0 | 100 (69.4%) | 97 (67.4%) |  | 87 (71.9%) | 86 (71.1%) |  |
| 1 | 44 (30.6%) | 47 (32.6%) |  | 34 (28.1%) | 35 (28.9%) |  |
| **Child Pugh grade** |  |  | 0.866 |  |  | 0.851 |
| Grade A | 123 (85.4%) | 122 (84.7%) |  | 105 (86.8%) | 104 (86.0%) |  |
| Grade B | 21 (14.6%) | 22 (15.3%) |  | 16 (13.2%) | 17 (14.0%) |  |
| **Targeted therapy** |  |  | 0.719 |  |  | 0.967 |
| None | 37 (25.7%) | 42 (29.2%) |  | 33 (27.3%) | 36 (29.8%) |  |
| Lenvatinib | 63 (43.8%) | 56 (38.9%) |  | 51 (42.1%) | 49 (40.5%) |  |
| Donafenib | 38 (26.4%) | 37 (25.7%) |  | 31 (25.6%) | 31 (25.6%) |  |
| Regorafenib | 6 (4.2%) | 9 (6.3%) |  | 6 (5.0%) | 5 (4.1%) |  |
| **Sessions of TACE** |  |  | 0.619 |  |  | 0.989 |
| 1 | 30 (20.8%) | 35 (24.3%) |  | 27 (22.3%) | 27 (22.3%) |  |
| 2 | 42 (29.2%) | 45 (31.3%) |  | 36 (29.8%) | 37 (30.6%) |  |
| ≥ 3 | 72 (50.0%) | 64 (44.4%) |  | 58 (47.9%) | 57 (47.1%) |  |
| **Viral infection** |  |  | 0.281 |  |  | >0.999 |
| Hepatitis B | 139 (96.5%) | 133 (92.4%) |  | 116 (95.9%) | 115 (95.0%) |  |
| Hepatitis C | 4 (2.8%) | 8 (5.6%) |  | 4 (3.3%) | 5 (4.1%) |  |
| Other | 1 (0.7%) | 3 (2.1%) |  | 1 (0.8%) | 1 (0.8%) |  |
| **Portal vein tumor thrombus** |  |  | 0.673 |  |  | 0.908 |
| None | 87 (60.4%) | 70 (48.6%) |  | 70 (57.9%) | 63 (52.1%) |  |
| Type Ⅰ | 21 (14.6%) | 21 (14.6%) |  | 17 (14.0%) | 19 (15.7%) |  |
| Type Ⅱ | 29 (20.1%) | 43 (29.9%) |  | 28 (23.1%) | 31 (25.6%) |  |
| Type Ⅲ | 6 (4.2%) | 9 (6.3%) |  | 5 (4.1%) | 7 (5.8%) |  |
| Type Ⅳ | 1 (0.7%) | 1 (0.7%) |  | 1 (0.8%) | 1 (0.8%) |  |
| **Ascites^d^** |  |  | 0.086 |  |  | >0.999 |
| None | 138 (95.8%) | 132 (91.7%) |  | 116 (95.9%) | 116 (95.9%) |  |
| Grade 1 | 6 (4.2%) | 9 (6.3%) |  | 5 (4.1%) | 5 (4.1%) |  |
| Grade 2 | 0 (0.0%) | 3 (2.1%) |  | 0 (0.0%) | 0 (0.0%) |  |
| **Albumin** |  |  | 0.105 |  |  | 0.670 |
| Mean (SD) | 36.2 (±4.8) | 35.3 (±4.6) |  | 35.9 (±4.7) | 35.8 (±4.6) |  |
| **ALT** |  |  | 0.676 |  |  | 0.585 |
| Median (IQR) | 54.0 (72.8) | 59.0 (65.8) |  | 50.9 (69.3) | 61.2 (71.6) |  |
| **AST** |  |  | 0.417 |  |  | 0.704 |
| Median (IQR) | 74.5 (95.0) | 60.5 (82.8) |  | 69.0 (77.4) | 59.3 (77.2) |  |
| **ALP** |  |  | 0.228 |  |  | 0.901 |
| Median (IQR) | 144.5 (129.3) | 167.0 (120.5) |  | 147.8 (141.2) | 153.0 (99.5) |  |
| **GGT** |  |  | 0.801 |  |  | >0.999 |
| Median (IQR) | 138.5 (165.0) | 131.0 (156.3) |  | 136.5 (156.4) | 130.0 (122.8) |  |
| **Platelet** |  |  | 0.362 |  |  | 0.955 |
| Median (IQR) | 127.0 (87.5) | 130 (105.3) |  | 129.1 (91.6) | 123.0 (96.0) |  |
| **PT** |  |  | 0.412 |  |  | 0.403 |
| Median (IQR) | 12.9 (1.9) | 13.10 (2.3) |  | 13.0 (2.0) | 13.0 (2.0) |  |
| **Total bilirubin** |  |  | 0.742 |  |  | 0.722 |
| Median (IQR) | 21.1 (13.7) | 20.2 (16.6) |  | 22.0 (11.2) | 20.3 (15.5) |  |
| **AFP** |  |  | 0.130 |  |  | 0.060 |
| Median (IQR) | 163.6 (990.7) | 58.9 (992.8) |  | 168.3 (990.3) | 43.1 (569.9) |  |
| **Extrahepatic metastases^e^** |  |  | 0.235 |  |  | >0.999 |
| No | 137 (95.1%) | 132 (91.7%) |  | 114 (94.2%) | 114 (94.2%) |  |
| Yes | 7 (4.9%) | 12 (8.3%) |  | 7 (5.8%) | 7 (5.8%) |  |
| **Number of lesions** |  |  | 0.238 |  |  | 0.775 |
| ≤ 3 | 46 (31.9%) | 35 (24.3%) |  | 35 (28.9%) | 33 (27.3%) |  |
| ＞3 | 98 (68.1%) | 109 (75.7%) |  | 86 (71.1%) | 88 (72.7%) |  |
| **Maximum diameter of lesion** |  |  | 0.960 |  |  | 0.809 |
| Median (IQR) | 6.2 (6.7) | 6.4 (6.6) |  | 6.1 (5.8) | 6.1 (5.6) |  |
| Abbreviations: Icaritin -TACE, transarterial chemoembolization plus Icaritin; TACE, transarterial chemoembolization; ECOG, Eastern Cooperative Oncology Group; ALT, alanine aminotransferase; AST, aspartate aminotransferase; ALP, Alkaline Phosphatase; GGT, gamma-glutamyl transferase; PT, prothrombin time (international ratio); AFP, alpha-Fetoprotein. ^a^n (%).^b^Pearson's Chi-squared test; Wilcoxon rank sum test; Fisher's exact test. ^c^ ECOG score of 0 indicates that patient is fully active and able to carry on all pre-disease activities without restriction, and 1 indicates that patient is restricted in physically strenuous activity but is ambulatory and able to carry out work of a light nature, including self-care. ^d^ Grade 1 indicates patients with mild ascites; Grade 2 indicates patients with moderate ascites. ^e^ Extrahepatic metastases include metastasis to one or more sites such as the lung, bone, and peritoneum. | | | | | | |
