# Supplementary material for: Icaritin plus TACE improves survival in advanced HCC with macrovascular invasion: a multicenter cohort study
Source: Front Immunol. 2026 May 29;17:1684486. doi: 10.3389/fimmu.2026.1684486 (PMC13260649; doi:10.3389/fimmu.2026.1684486)
Supplement: Supplementary file 9 [file Table5.docx]

| **Supplementary Table 5. Best Tumor Response Following Treatment with Icaritin–TACE Versus TACE Alone after Propensity Score Matching** | | | |
| --- | --- | --- | --- |
| **Response Category** | **Icaritin-TACE group (n = 121)** | **TACE group (n = 121)** | ***P*** |
| **Complete response** | **14 (11.6%)** | **10 (8.3%)** | **0.390** |
| **Partial response** | **53 (43.8%)** | **48 (39.7%)** | **0.515** |
| **Stable disease** | **33 (27.3%)** | **32 (26.4%)** | **0.885** |
| **Progressive disease** | **21 (17.4%)** | **34 (28.1%)** | **0.046** |
| **Objective response rate** | **67 (55.4%)** | **58 (47.9%)** | **0.247** |
| **Disease control rate** | **103 (85.1%)** | **90 (74.4%)** | **0.038** |

Abbreviations: Icaritin -TACE, transarterial chemoembolization plus Icaritin; TACE, transarterial chemoembolization. The objective response rate was defined as the proportion of patients with complete response plus partial response. The disease control rate was defined as the proportion of patients with complete response plus partial response and stable disease. P values calculated by Chi-square test.
