# Supplementary material for: Icaritin plus TACE improves survival in advanced HCC with macrovascular invasion: a multicenter cohort study
Source: Front Immunol. 2026 May 29;17:1684486. doi: 10.3389/fimmu.2026.1684486 (PMC13260649; doi:10.3389/fimmu.2026.1684486)
Supplement: Supplementary file 10 [file Table6.docx]

| **Supplementary Table 6. Univariate and Multivariate COX Analysis of Overall Survival as Sensitivity Analysis after Propensity Score Matching** | | | | | | | |
| --- | --- | --- | --- | --- | --- | --- | --- |
| **Characteristic** | **Univariable** | | | | **Multivariable** | | |
|  | **HR** | **95% CI** | ***P*** | **HR** | | **95% CI** | ***P*** |
| **Gender** |  |  |  |  | |  |  |
| Male | — | — |  |  | |  |  |
| Female | 1.05 | 0.67, 1.65 | 0.823 |  | |  |  |
| **Age** |  |  |  |  | |  |  |
| ＜50 yrs | — | — |  |  | |  |  |
| ≥ 50 yrs | 1.00 | 0.75, 1.33 | 0.986 |  | |  |  |
| **ECOG score** |  |  |  |  | |  |  |
| 0 | — | — |  | — | | — |  |
| 1 | 1.90 | 1.42, 2.54 | <0.001 | 1.43 | | 1.01, 2.02 | 0.041 |
| **Child Pugh grade** |  |  |  |  | |  |  |
| Grade A | — | — |  | — | | — |  |
| Grade B | 1.24 | 0.85, 1.81 | 0.263 | 1.04 | | 0.69, 1.58 | 0.839 |
| **Targeted therapy** |  |  |  |  | |  |  |
| None | — | — |  | — | | — |  |
| Lenvatinib | 0.95 | 0.68, 1.32 | 0.753 | 0.78 | | 0.55, 1.11 | 0.169 |
| Donafenib | 1.23 | 0.85, 1.78 | 0.274 | 1.08 | | 0.73, 1.58 | 0.711 |
| Regorafenib | 1.79 | 0.94, 3.41 | 0.079 | 1.72 | | 0.86, 3.45 | 0.125 |
| **Sessions of TACE** |  |  |  |  | |  |  |
| 1 | — | — |  |  | |  |  |
| 2 | 1.01 | 0.69, 1.46 | 0.979 |  | |  |  |
| ≥ 3 | 1.09 | 0.77, 1.55 | 0.625 |  | |  |  |
| **Viral infection** |  |  |  |  | |  |  |
| Hepatitis B | — | — |  | — | | — |  |
| Hepatitis C | 1.18 | 0.58, 2.39 | 0.650 | 1.66 | | 0.80, 3.48 | 0.176 |
| Other | 4.51 | 1.10, 18.48 | 0.036 | 3.25 | | 0.74, 14.22 | 0.117 |
| **Portal vein tumor thrombus^a^** |  |  |  |  | |  |  |
| None | — | — |  | — | | — |  |
| Type Ⅰ | 2.14 | 1.43, 3.20 | <0.001 | 2.39 | | 1.55, 3.67 | <0.001 |
| Type Ⅱ | 3.25 | 2.33, 4.52 | <0.001 | 3.32 | | 2.29, 4.81 | <0.001 |
| Type Ⅲ | 6.10 | 3.35, 11.11 | <0.001 | 6.02 | | 3.06, 11.83 | <0.001 |
| Type Ⅳ | 2.34 | 0.57, 9.59 | 0.236 | 3.13 | | 0.71, 13.86 | 0.133 |
| **Ascites^b^** |  |  |  |  | |  |  |
| None | — | — |  | — | | — |  |
| Grade 1 | 2.57 | 1.25, 5.26 | 0.010 | 2.06 | | 0.90, 4.71 | 0.087 |
| Grade 2 |  |  |  |  | |  |  |
| **AFP** | — | — |  | — | | — |  |
| ＜400 ng/mL | 0.80 | 0.60, 1.06 | 0.119 | 0.75 | | 0.55, 1.02 | 0.068 |
| ≥ 400 ng/mL |  |  |  |  | |  |  |
| **Extrahepatic metastases** | — | — |  | — | | — |  |
| No | 1.83 | 1.04, 3.21 | 0.036 | 0.88 | | 0.44, 1.75 | 0.712 |
| Yes |  |  |  |  | |  |  |
| **Number of lesions** | — | — |  | — | | — |  |
| ≤ 3 | 1.97 | 1.44, 2.70 | <0.001 | 1.51 | | 1.08, 2.13 | 0.017 |
| ＞3 |  |  |  |  | |  |  |
| **Maximum diameter of lesion** | — | — |  | — | | — |  |
| ＜5 cm | 0.71 | 0.54, 0.93 | 0.013 | 0.59 | | 0.43, 0.81 | 0.001 |
| ≥ 5 cm |  |  |  |  | |  |  |
| Abbreviations: CI, Confidence Interval; HR, Hazard Ratio; ECOG, Eastern Cooperative Oncology Group; AFP, Alpha-Fetoprotein. ^a^ According to the Cheng's classification (Cheng Shuqin classification) used in China. Type Ⅰ: the tumor thrombus is located in the portal vein branches of the hepatic segment or subsegment; Type Ⅱ: tumor thrombus invades the left or right branch of the portal vein; Type Ⅲ: tumor thrombus involves the main trunk of the portal vein; Type Ⅳ: tumor thrombus extends into the superior mesenteric vein or splenic vein. ^b^ Grade 1 indicates patients with mild ascites; Grade 2 indicates patients with moderate ascites. | | | | | | | |
