# Supplementary material for: Icaritin plus TACE improves survival in advanced HCC with macrovascular invasion: a multicenter cohort study
Source: Front Immunol. 2026 May 29;17:1684486. doi: 10.3389/fimmu.2026.1684486 (PMC13260649; doi:10.3389/fimmu.2026.1684486)
Supplement: Supplementary file 11 [file Table7.docx]

| **Supplementary Table 7. Univariate and Multivariate COX Analysis of Progression-free Survival as Sensitivity Analysis after Propensity Score Matching** | | | | | | | |
| --- | --- | --- | --- | --- | --- | --- | --- |
| **Characteristic** | **Univariable** | | | | **Multivariable** | | |
|  | **HR** | **95% CI** | ***P*** | **HR** | | **95% CI** | ***P*** |
| **Gender** |  |  |  |  | |  |  |
| Male | — | — |  |  | |  |  |
| Female | 0.83 | 0.54, 1.27 | 0.395 |  | |  |  |
| **Age** |  |  |  |  | |  |  |
| ＜50 yrs | — | — |  |  | |  |  |
| ≥ 50 yrs | 0.88 | 0.67, 1.16 | 0.363 |  | |  |  |
| **ECOG score** |  |  |  |  | |  |  |
| 0 | — | — |  | — | | — |  |
| 1 | 1.51 | 1.14, 2.00 | 0.004 | 1.33 | | 0.99, 1.80 | 0.062 |
| **Child Pugh grade** |  |  |  |  | |  |  |
| Grade A | — | — |  |  | |  |  |
| Grade B | 1.03 | 0.72, 1.50 | 0.856 |  | |  |  |
| **Targeted therapy** |  |  |  |  | |  |  |
| None | — | — |  | — | | — |  |
| Lenvatinib | 1.04 | 0.76, 1.42 | 0.821 | 0.94 | | 0.68, 1.30 | 0.704 |
| Donafenib | 1.27 | 0.90, 1.81 | 0.178 | 1.20 | | 0.83, 1.71 | 0.331 |
| Regorafenib | 1.91 | 1.01, 3.64 | 0.048 | 1.82 | | 0.93, 3.56 | 0.081 |
| **Sessions of TACE** |  |  |  |  | |  |  |
| 1 | — | — |  |  | |  |  |
| 2 | 1.24 | 0.86, 1.79 | 0.254 |  | |  |  |
| ≥ 3 | 1.12 | 0.80, 1.58 | 0.501 |  | |  |  |
| **Viral infection** |  |  |  |  | |  |  |
| Hepatitis B | — | — |  |  | |  |  |
| Hepatitis C | 1.16 | 0.57, 2.34 | 0.688 |  | |  |  |
| Other | 1.25 | 0.31, 5.04 | 0.754 |  | |  |  |
| **Portal vein tumor thrombus^a^** |  |  |  |  | |  |  |
| None | — | — |  | — | | — |  |
| Type Ⅰ | 1.76 | 1.20, 2.60 | 0.004 | 1.72 | | 1.16, 2.55 | 0.007 |
| Type Ⅱ | 1.89 | 1.39, 2.58 | <0.001 | 1.72 | | 1.25, 2.36 | <0.001 |
| Type Ⅲ | 2.97 | 1.66, 5.32 | <0.001 | 2.81 | | 1.51, 5.24 | 0.001 |
| Type Ⅳ | 1.82 | 0.45, 7.42 | 0.401 | 1.57 | | 0.36, 6.83 | 0.546 |
| **Ascites^b^** |  |  |  |  | |  |  |
| None | — | — |  | — | | — |  |
| Grade 1 | 2.06 | 1.05, 4.03 | 0.035 | 1.33 | | 0.63, 2.82 | 0.450 |
| Grade 2 |  |  |  |  | |  |  |
| **AFP** | — | — |  |  | |  |  |
| ＜400 ng/mL | 0.89 | 0.68, 1.16 | 0.392 |  | |  |  |
| ≥ 400 ng/mL |  |  |  |  | |  |  |
| **Extrahepatic metastases** | — | — |  |  | |  |  |
| No | 1.59 | 0.92, 2.73 | 0.095 |  | |  |  |
| Yes |  |  |  |  | |  |  |
| **Number of lesions** | — | — |  | — | | — |  |
| ≤ 3 | 1.58 | 1.18, 2.11 | 0.002 | 1.36 | | 1.00, 1.83 | 0.048 |
| ＞3 |  |  |  |  | |  |  |
| **Maximum diameter of lesion** | — | — |  |  | |  |  |
| ＜5 cm | 0.89 | 0.68, 1.16 | 0.385 |  | |  |  |
| ≥ 5 cm |  |  |  |  | |  |  |
| Abbreviations: CI, Confidence Interval; HR, Hazard Ratio; ECOG, Eastern Cooperative Oncology Group; AFP, Alpha-Fetoprotein. ^a^ According to the Cheng's classification (Cheng Shuqin classification) used in China. Type Ⅰ: the tumor thrombus is located in the portal vein branches of the hepatic segment or subsegment; Type Ⅱ: tumor thrombus invades the left or right branch of the portal vein; Type Ⅲ: tumor thrombus involves the main trunk of the portal vein; Type Ⅳ: tumor thrombus extends into the superior mesenteric vein or splenic vein. ^b^ Grade 1 indicates patients with mild ascites; Grade 2 indicates patients with moderate ascites. | | | | | | | |
