# Supplementary material for: Icaritin plus TACE improves survival in advanced HCC with macrovascular invasion: a multicenter cohort study
Source: Front Immunol. 2026 May 29;17:1684486. doi: 10.3389/fimmu.2026.1684486 (PMC13260649; doi:10.3389/fimmu.2026.1684486)
Supplement: Supplementary file 12 [file Table8.docx]

| **Supplementary Table 8. Variance Inflation Factor and Tolerance** | | | | | | | |
| --- | --- | --- | --- | --- | --- | --- | --- |
| **Term** | **VIF** | **VIF CI low** | **VIF CI high** | **SE factor** | **Tolerance** | **Tolerance CI low** | **Tolerance CI high** |
| Age | 1.11406 | 1.036383 | 1.357574 | 1.05549 | 0.897618 | 0.736608 | 0.964894 |
| ECOG score | 1.430514 | 1.272121 | 1.681102 | 1.196041 | 0.699049 | 0.594848 | 0.786089 |
| Child Pugh grade | 1.216477 | 1.107236 | 1.436999 | 1.10294 | 0.822046 | 0.695895 | 0.90315 |
| Targeted therapy | 1.510515 | 1.335397 | 1.777066 | 1.22903 | 0.662026 | 0.562725 | 0.748841 |
| Sessions of TACE | 1.250862 | 1.132906 | 1.473503 | 1.118419 | 0.799449 | 0.678655 | 0.882686 |
| Viral infection | 1.991403 | 1.720427 | 2.364302 | 1.411171 | 0.502159 | 0.422958 | 0.581251 |
| Portal vein tumor thrombus | 2.040826 | 1.760195 | 2.425053 | 1.428575 | 0.489998 | 0.412362 | 0.568119 |
| Ascites | 2.786933 | 2.361894 | 3.344624 | 1.669411 | 0.358817 | 0.298987 | 0.423389 |
| AFP | 1.184253 | 1.083775 | 1.405244 | 1.088234 | 0.844414 | 0.71162 | 0.922701 |
| Extrahepatic metastases | 1.264548 | 1.143258 | 1.488527 | 1.124521 | 0.790797 | 0.671805 | 0.874693 |
