# Supplementary material for: Icaritin plus TACE improves survival in advanced HCC with macrovascular invasion: a multicenter cohort study
Source: Front Immunol. 2026 May 29;17:1684486. doi: 10.3389/fimmu.2026.1684486 (PMC13260649; doi:10.3389/fimmu.2026.1684486)
Supplement: Supplementary file 13 [file Table9.docx]

| Supplementary Table 9. Adverse Events | | | | | | |
| --- | --- | --- | --- | --- | --- | --- |
|  | Any grade, n (%) | | | Grade 3-4, n(%) | | |
| Event | Icaritin-TACE (n = 144) | TACE alone (n = 144) | *P* | Icaritin-TACE (n = 144) | TACE alone (n = 144) | *P* |
| Total bilirubin increased | 128 (88.9) | 124 (86.1) | 0.472 | 28 (19.4) | 23 (16.0) | 0.382 |
| GGT increased | 112 (77.8) | 118 (81.9) | 0.375 | 3 (2.1) | 2 (1.4) | 0.652 |
| ALT increased | 135 (93.8) | 130 (90.3) | 0.257 | 19 (13.2) | 20 (13.9) | 0.858 |
| AST increased | 122 (84.7) | 118 (81.9) | 0.527 | 15 (10.4) | 13 (9.0) | 0.683 |
| Diarrhea | 78 (54.2) | 31 (21.5) | <0.001 | 6 (4.2) | 4 (2.8) | 0.504 |
| PT prolonged | 56 (38.9) | 49 (34.0) | 0.398 | 2 (1.4) | 1 (0.7) | 0.622 |
| Hypoalbuminemia | 82 (56.9) | 69 (47.9) | 0.123 | 5 (3.5) | 3 (2.1) | 0.477 |
| Hand-footskinreaction | 15 (10.4) | 20 (13.9) | 0.355 | 3 (2.1) | 4 (2.8) | 0.703 |
| Ascites worsening | 31 (21.5) | 34 (23.6) | 0.669 | 3 (2.1) | 2 (1.4) | 0.652 |
| Abdominal pain | 125 (86.8) | 128 (88.9) | 0.589 | 34 (23.6) | 45 (31.3) | 0.122 |
| Hepatic encephalopathy | 14 (9.7) | 11 (7.6) | 0.523 | 3 (2.1) | 2 (1.4) | 0.652 |
| Gastrointestinal bleeding | 14 (9.7) | 10 (6.9) | 0.386 | 4 (2.8) | 2 (1.4) | 0.408 |
| Nausea | 116 (80.6) | 108 (75.0) | 0.245 | 25 (17.4) | 22 (15.3) | 0.622 |
| Thrombocytopenia | 94 (65.3) | 102 (70.8) | 0.305 | 12 (8.3) | 13 (9.0) | 0.836 |
| Albuminuria | 34 (23.6) | 21 (14.6) | 0.049 | 1 (0.7) | 0 (0) | 0.317 |
| Pruritus | 13 (9.0) | 14 (9.7) | 0.836 | 2 (1.4) | 2 (1.4) | 1.000 |
| Fever | 89 (61.8) | 94 (65.3) | 0.536 | 26 (18.1) | 22 (15.3) | 0.527 |
| Weight decreased | 56 (38.9) | 47 (32.6) | 0.267 | 3 (2.1) | 2 (1.4) | 0.652 |
| Anemia | 37 (25.7) | 34 (23.6) | 0.677 | 5 (3.5) | 7 (4.9) | 0.527 |
| Hypertension | 23 (16.0) | 11 (7.6) | 0.027 | 6 (4.2) | 4 (2.8) | 0.408 |
| ECG T-wave abnormalities | 12 (8.3) | 8 (5.6) | 0.355 | 0 (0) | 0 (0) | -† |
| WBC count decreased | 32 (22.2) | 29 (20.1) | 0.669 | 2 (1.4) | 1 (0.7) | 0.622 |
| Electrolyte imbalance | 32 (22.2) | 26 (18.1) | 0.386 | 5 (3.5) | 2 (1.4) | 0.252 |
| Dysgeusia | 2 (1.4) | 1 (0.7) | -.562 | 0 (0) | 0 (0) | -† |

Abbreviations: Icaritin-TACE, transarterial chemoembolization plus Icaritin; TACE, transarterial chemoembolization; GGT, gamma-glutamyl transferase; ALT, alanine aminotransferase; AST, aspartate aminotransferase; ECG, electrocardiograph; WBC, white blood cell; PT, prothrombin time. P values calculated by Chi-square test (or Fisher’s exact test if expected cell count <5). †: Not applicable due to zero events in both groups for Grade 3-4.
